# Greater Alpine river network evolution, interpretations based on novel drainage analysis

**DOI:** 10.1007/s00015-018-0332-5

**Published:** 2018-11-22

**Authors:** Sascha Winterberg, Sean D. Willett

**Affiliations:** Geological Institute, ETH Zürich, 8092 Zurich, Switzerland

**Keywords:** Alps, Danube river, Geodynamics, Geomorphology, χ-map

## Abstract

The Central European drainage system is dominated by four major rivers (the Danube, Rhine, Rhône and Po). The geometry of these drainage basins has evolved through the history of Alpine and Carpathian orogeny. Analysis of the modern river geometry reveals the geometric stability or instability of the drainage network and enables interpretation of the erosion and exhumation pattern. We characterize the river basin geometry and inter-basin relief through metrics including the quantity χ that relates to the catchment area, and a catchment restricted minimum elevation (CRM) metric. The interpretations from the maps are in agreement with known river captures and morphological features. The χ-map reveals additional systematic, large-scale transients, consistent with ongoing basin changes, mainly manifesting in the Danube losing catchment area. We postulate that the changes are related to the longitudinal initiation of the Danube River in the Alpine foreland, augmented by the formation of the Carpathians and the filling of the Pannonian Basin, which resulted in an elongation of the Danube river basin. We conclude that the Danube has lacked erosional power throughout its history and therefore been victim to capture and area loss.

**Electronic supplementary material:**

The online version of this article (10.1007/s00015-018-0332-5) contains supplementary material, which is available to authorized users.

## Introduction

### Scope

**Fig. 1:**
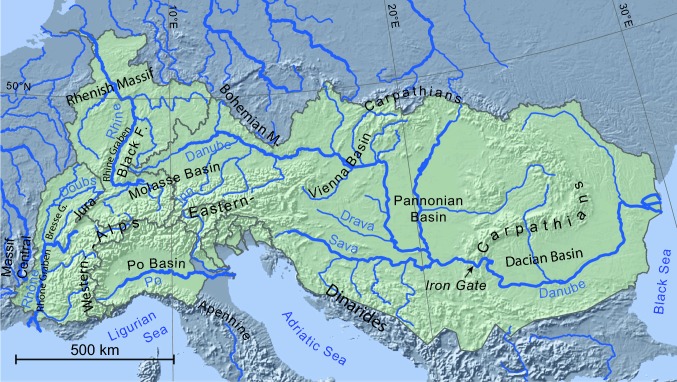
Map of Europe with catchment outlines (dark grey) of rivers draining the Alps. The Rhine, Rhône, Po and Danube Rivers are the main drainage basins of the Alps. In the case of the Danube, the river drains areas far away from the Alps, crossing the Carpathian Mountains at the Iron Gate. Map projection is WGS84 UTM 32 N

The major European river basins that define the prominent elements of the continental geography reflect the geodynamic history of the region. The Alpine mountain range is the prime example of this control, defining the continental divide and providing the headwaters of the major rivers of Europe (Fig. 1). The formation of the Alpine belt over the last 30 My established the water divides and catchments of the major rivers draining the mountains into the surrounding basins (Kuhlemann 2007), and ultimately into the major water bodies north, south, west and east of the continent. The large-scale physical geography of the Alps reflects both the collision-tectonic and post-collisional phases of deformation and uplift, with important changes in drainage over time.

In this paper, we analyse rivers of the Alpine chain and surrounding regions where base level changes and tectonic activity also affect river basin geometry. We use drainage basin topology and geometry to map geometric asymmetry and thus transience (Willett et al. 2014). We also use a new tool, *catchment restricted minimum altitude* (CRM) analysis, to complement the geomorphic analysis and investigate the current state of the river network. Much of the geologic history of the continent is evident in this drainage basin structure. We document past events, the current state, and make predictions about the future evolution of the drainage network over geologic timescales. The resulting maps reveal large-scale interactions between the major river basins and allow a new view of European geographic evolution. We demonstrate that the current examples of drainage divide migration are systematic and governed by the large-scale basin geometries and interactions amongst them. We discuss the particular network geometry of the Danube and its effect on Alpine river basin transients. This holistic drainage network analysis reveals new geodynamic interactions between the major river basins and the Alps.

### River network and geography

The modern Po basin drains the southern flank of the Alps and flows longitudinally to the east within the southern foreland basin. The modern Northern Alpine Foreland Basin (NAFB) is partitioned between several major river basins, including the Rhône, Rhine and Danube. In the early basin evolution, a Paleo-Rhône river drained the entire foreland, from the Mediterranean to the Bohemian Massive, transporting water and sediment to the west into the Bresse graben (Ziegler and Fraefel 2009). Late Alpine (late Miocene) Jura thrusting reversed the flow direction in the NAFB from west to east, where the sediments transported by the Danube filled the Pannonian basin, which was the base level for this system since at least the Miocene (Kuhlemann and Kempf 2002; Ziegler and Fraefel 2009).

The Danube has a particularly complex history, amongst other factors also reflecting the base level change associated with a regression of the sea to the east, as the Paratethys Sea retreated to its current remnants in the Black Sea (Hámor 1988; Popov et al. 2006). This regression elongated the path of the river, ultimately producing a long, relatively narrow drainage basin. Moreover, the growth of the Carpatho-Balkan chain created a barrier to the Danube River basin, which separated the Pannonian Basin from the Paratethys at the Iron Gate in the Carpathians (location in Fig. 1), although the timing of the connection for both water and sediment remains contentious (Olariu et al. 2017).

Similarly, the Rhine was affected by significant late Cenozoic tectonic events, primarily the opening and subsidence of the Rhine Graben system (Schumacher 2002; Madritsch et al. 2010; Schlunegger and Mosar 2010). The Bresse and Rhine Graben are both part of the reactivated European Cenozoic Rift System (ECRIS) that opened a new, efficient drainage path towards the Rhône (e.g. Dèzes et al. 2004 and references therein). After the Pliocene, the Rhine offered an even shorter path to base level, and eventually captured the Aare-Rhône (Laubscher 2001; Giamboni et al. 2004), establishing the modern drainage for much of the NAFB and the Alps that is still expanding today as we will demonstrate.

Paleo-geographic reconstructions have addressed the evolution of paleo-drainage through a synthesis of geologic observations. Handy et al. (2014) restored the general plate tectonic movements using structural evidence, cross section restoration and geophysical observations in the Alps. Ustaszewski et al. (2008) completed a kinematic analysis to reconstruct the Carpathian evolution. Fission track analysis and provenance analysis have been used to establish sediment pathways in the Eastern Alps (Frisch et al. 1998; Dunkl et al. 2005). Detailed analysis of river gravel and surface morphology have been integrated to restore the Danube and Rhine river course in the Bavarian and Swabian areas (Villinger 1998). Kuhlemann (2007) used sediment budgets from around the Alps and converted them to erosion volumes thereby inferring river basin extent and erosion rates on a continental scale. Further to the east, marine facies analysis in the Paratethys enabled Popov et al. (2006) and later Palcu et al. (2015) to reconstruct the extent of the sea in the Pannonian and Black Sea region, providing important constraints on coastal retreat and the changing drainage area. Seismic section interpretation revealed a detailed image of the progradation of the shelf in the Pannonian basin (Magyar et al. 2013). Similar reconstructions based on sediments were done for the Po Basin and Adriatic Sea (Fantoni and Franciosi 2010; Ghielmi et al. 2010).

The aforementioned dynamic changes to the drainage patterns of the European continent imply a large transience in the hydro-geomorphic system. An important remaining question is how much of this transience remains today. Given that Alpine convergent tectonism has largely ended, is the drainage system evolution completed? Or do we see evidence for ongoing change in the modern system?

Geomorphic analysis of modern river basins shows evidence of the complex geologic history of the Alps, and has illuminated the current state of dynamic basin processes. River profiles illustrate recent changes in river channel topology and the subsequent erosional responses that propagated up and down along the river network (Yanites et al. 2013). Evaluation of channel steepness and drainage basin geometry shows rivers draining to the south in the Eastern Alps have expanded towards the north by drainage divide migration and river capture (Robl et al. 2017). By utilizing the χ-mapping techniques of Willett et al. (2014), they demonstrated that most of the drainages in the eastern Alps appear unstable in their geometry. Robl et al. (2017) attributed this transience to the Messinian sea level fall and a base level rise in the Molasse basin. Other models explain these and other instabilities as a response to strike-slip faulting activity (Bartosch et al. 2017).

Desiccation of the Mediterranean Sea in the Messinian caused a sea level fall and led to the deposition of salt in marine basins (Hsü et al. 1973), the Messinian Salinity Crisis (MSC) was short lived in the late Messinian stage. The rivers incised deeply, channel geometry could not reach steady state in this short period of time and therefore preserved a convex form (Clauzon 1982). Incisions are reported in the Rhône Valley, and the rivers draining to the Po Plain and the Adriatic Sea (Clauzon 1982; Ghielmi et al. 2010).

Climate is another factor potentially affecting the river network. The climatic conditions in the last 5 My showed high variability, including ice ages (Bini et al. 2009). Precipitation changes in the past affected the areas on a large scale hence had small influence on a local scale. Glacial perturbations strongly modified the topography, although restricted to the ice extent (see Bini et al. 2009). The glacial moraines have actively diverted rivers, but detailed timing of these events is not known (Villinger 1998). Recent glacial retreat is taking place within the high Alps.

### Tectonic and geologic setting

The Alps are part of a convergent orogenic belt between continental Europe and southerly continental plates or micro plates. Subduction of European lithosphere below the Adriatic lithosphere was the main driver for the Alpine orogeny from 70 to 35 Ma (Schmid et al. 2004; Handy et al. 2014). After 35 Ma, buoyant European crust entered the subduction zone and initiated crustal thickening with isostatic surface uplift, during the main Alpine collision (Schmid et al. 1996; Handy et al. 2014).

Cretaceous (Eo-Alpine) top-WNW thrusting in the Eastern Alps, followed by early Cenozoic top-N collision in the Central and Eastern Alps (Froitzheim et al. 1994), and late Oligocene to early Miocene top-NNW thrusting in the Western Alps (Schmid et al. 2017) formed the present-day Alps. The Miocene Jura thrusting and folding induced coeval uplift of the Molasse in Switzerland as a piggyback basin (Kuhlemann and Kempf 2002), extending elevated topography to the north. Until today, Alpine convergence rates decreased strongly. In the Central and Western Alps convergence has ceased (Calais et al. 2002; Sue et al. 2007), in the Eastern Alps convergence is continuing, largely taken up by thrusting on the southern Alpine border regions (Danesi et al. 2015). Lateral extrusion associated with strike slip faulting and orogen-parallel extrusion was active until at least the Miocene (Ratschbacher et al. 1991; Linzer et al. 2002).

Current deformation in the Western and Central Alps is largely limited to vertical motions in response to erosional unloading or slab break-off and restoration of gravitational equilibrium by extension (Sue et al. 2007; Fox et al. 2015). Although paleo-elevation estimates are difficult to ascertain, isotopic data and fault geometries suggest that the elevation of the Alps were at their maximum or slightly above modern levels (around 2300 m a.s.l.) in the Miocene and maintained this elevation until today (Kuhlemann 2007; Campani et al. 2012; Schlunegger and Kissling 2015; Dielforder 2017). This is consistent with arguments that the orogenic wedge reached its maximum active extent and elevation during Jura thrusting in the latest Miocene (Willett et al. 2006). The long-term exhumation rate of the Alps is best characterized by thermochronometric data, a summary and interpretation was given by Fox et al. (2016). A more local analysis for the exhumation for the Molasse was assessed by Cederbom et al. (2004). Miocene to recent changes of topography are of minor magnitude and are a topic of ongoing research (Willett et al. 2006; Schlunegger and Kissling 2015). Relief is possibly higher today even though similar average elevations have been maintained (Champagnac et al. 2007). Furthermore, the foreland was inverted from subsiding and depositional to uplifting and erosional conditions due to orogenic rebound (Cederbom et al. 2004).

In the eastern Alps, lateral eastward extrusion and exhumation of the Tauern Window starting in the early Miocene is the result of orogen-perpendicular shortening and orogen-parallel extension associated with motion on strike-slip faults (Ratschbacher et al. 1991; Linzer et al. 2002). Lateral extrusion was facilitated by subduction and rollback of the Carpathian slabs with respect to central Europe and the Alps (Ustaszewski et al. 2008; Scharf et al. 2013). Faulting rerouted rivers in the Eastern Alps along strike slip faults towards the east (Kuhlemann and Kempf 2002; Bartosch et al. 2017).

The Pannonian Basin is an extensional basin that formed as a consequence of the rollback of the Carpathian slab (Ustaszewski et al. 2008; Balázs et al. 2016). As a physiographic feature, its continued existence as a sediment trap is aided by the barrier of the Carpathian range. The Carpathians were tectonically active through most of the Miocene (Kováč et al. 2017), while tectonic activity in the southern edge of the East Carpathians continued into the Pliocene (Matenco et al. 2003; Necea et al. 2013). Due to the Carpathian uplift, the Pannonian Basin was isolated from the Eastern Paratethys around 12.6 Ma. Lake Pannon reached its maximum extent after isolation (ter Borgh et al. 2013; Palcu et al. 2015). Fluctuating lake levels indicate a closed basin (Magyar et al. 2013; Balázs et al. 2016), but a fluvial connection across the iron gate cannot be excluded, possibly as overspill events (Leever et al. 2011). After the Messinian a permanent fluvial system across the Carpathians is present (Leever et al. 2011).

The Apennines on the Italian peninsula remained more active in Pliocene times, in contrast to the Alpine or Carpathian ranges to the north (Molli et al. 2010; Schmid et al. 2017). This tectonic activity is driven by ongoing subduction of Adria to the west below the Apennines. The Adriatic slab below the Apennines is rolling back, leading to the formation of the Tyrrhenian Sea back-arc basin (Royden and Faccenna 2018 and references therein). At the surface, this rollback forces the high topography to migrate to the NE with respect to Europe and Adria, also compression and extension migrate spatially with time (Mazzanti and Trevisan 1978). The Po basin serves as the pro-foreland basin to the Apennines and the retro-foreland basin to the Alps (Ghielmi et al. 2010; Handy et al. 2010). As a physiographic feature, the Po basin is closed on three sides, forcing sediment from both the Alps and the Apennines down the basin axis to the Adriatic Sea, resulting in overfilling of the upper basin and thereby bringing it above sea level (Ghielmi et al. 2010).

After the end of the thin-skinned folding and thrusting in the Jura Mountains north of the Western Alps at around 5 Ma (Ustaszewski and Schmid 2007) and after the decline of back thrusting of the Southern Alps during the Messinian (Schönborn 1992; Willett et al. 2006), the Alps entered a post-collisional stage where co-evolution of the tectonics and geomorphology has changed. The absence of significant convergence between Adria and Europe resulted in a stage where slab processes and surface denudation with mass removal became relatively more important (Willett et al. 2006; Schlunegger and Mosar 2010).

## Geomorphic analysis and methodology

The Alps offer one of the best opportunities to conduct large-scale analysis of the surface response to orogenesis and its cessation, due to the extensive geological knowledge. In order to investigate the current state of the topography we took advantage of the available elevation models and used them to analyse catchment geometry and cross-catchment elevation, augmenting these with topographic profiles. These results expand the basis for an interpretation of the landscape evolution over the course of the Alpine history.

### Topographic analysis

The surface analysis is based on the Shuttle Radar Topography Mission (SRTM) dataset from the United States Geological Survey (USGS) (3” resolution from earthexplorer.usgs.gov). This elevation model was checked to be hydrologically correct at a large scale in order to calculate flow directions defining the topology of the river network. Sinks and errors in river courses were corrected in order to get an accurate river flow direction for each cell in the model. In alluvial plains, the data tend to be more susceptible to unknown flow directions or direct human influence on the landscape. Therefore, in plains or flat areas of lakes, the elevation model is somewhat arbitrary, but provided the large-scale drainage areas are accurately defined, small elevation errors do not strongly affect our analysis.

### River basin analysis

Using aspects of the river drainage basin geometry is a well-established way to study a landscape (e.g. Hack 1973). The river basin offers two key metrics to analyse surface topography: the river elevation profile and the river catchment area and its distribution along the trunk channel. These metrics enable interpretation of perturbations due to tectonic or climate conditions (Whipple 2009). Profile analysis is best to reveal the evolution of a single river, whereas large-scale analysis of river networks offers insights into dynamics and interactions between drainage basins such as the expansion or shrinking of catchment area.

**Fig. 2:**
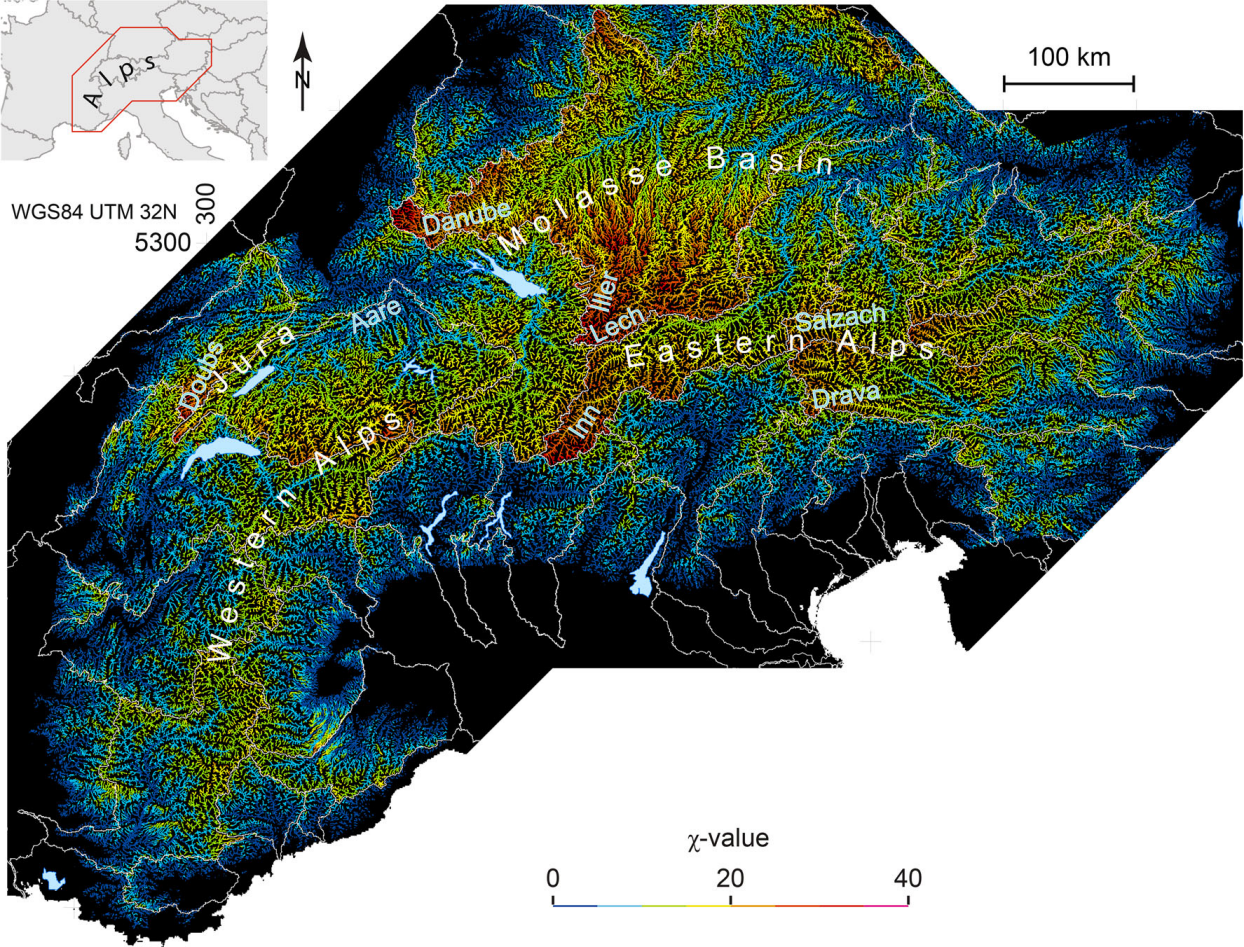
Alpine χ-map with a base level of 250 m a.s.l. White lines refer to the main river catchment outlines. Values of χ can be interpreted as expected elevation at equilibrium under constant uplift and climate conditions (see text). Very high χ-values are observed in the headwaters of the Inn and the Lech, which are the major Alpine tributaries of the Danube

We explore the river network by studying deviations from a geometrical steady state of all river profiles in a given area using a χ-map (Willett et al. 2014). This method shows the geometric deviation from steady state (Fig. 2). This analysis is based on the relationship between channel slope and catchment area, which scale linearly in log–log space above a threshold area in steady state between uplift and erosion (Whipple 2004; Wobus et al. 2006). Integration of this relationship implies a linear relationship between elevation and the integral quantity, χ (Perron and Royden 2013; Willett et al. 2014), defined as, $\chi = \mathop \smallint \limits_{{x_{b} }}^{x} \left( {\frac{{A_{0} }}{{A\left( {x'} \right)}}} \right)^{{\frac{m}{n}}} dx'$. In our calculation, the scaling area, A_0_ is set to 1 m^2^ and the concavity, $\frac{m}{n}$ is set to 0.45 (see Perron and Royden 2013). The base level *x*_*b*_ is chosen to be identical for all rivers. The elevation of $x_{b} =$ 250 m a.s.l. is used because it is the elevation where the Danube and the Rhine leave the Alpine domain. Resulting χ-values can be sensitive to the selected base level elevation (Forte and Whipple 2018; Giachetta and Willett 2018), so we investigate and discuss this sensitivity in the interpretation (see 4.1 and Online Resource 1). For every cell, we calculated the upstream catchment area *A*(*x*^’^) with a flow accumulation approach. In order to avoid the areas where hillslope processes dominate, we define a minimum area to form a channelized network, using a value of 1 km^2^. The calculation was done with the Matlab TopoToolbox (Schwanghart and Scherler 2014).

### Hillslope processes

Hillslope processes dominate the area above a specified threshold area to form a river. There is no permanent drainage network, and slopes depend on lithology, water storage time and other parameters. We assume no stream network for catchments below one square kilometre. Landslides and other processes transport material to river channels. We assume that the long-term hillslope erosion rates are governed by the river down-cutting rates, and slopes maintain an equilibrium with the river incision rate of the channel in which they drain.

### χ-Mapping and presentation of χ-values in terms of catchment

The resulting Alpine χ-map shows the river network in colour coding according to the χ-value of the river segment (Fig. 2), where low values are shown in blue and high values in red. Rivers in equilibrium with constant forcing and physical properties should have identical values for the same elevation. In particular, if neighbouring catchments exhibit different χ-values across common divides, this can be interpreted as an indication of transience in the geometry of the drainage basin (Willett et al. 2014; Forte and Whipple 2018). This will be discussed in depth in the interpretation section.

**Fig. 3:**
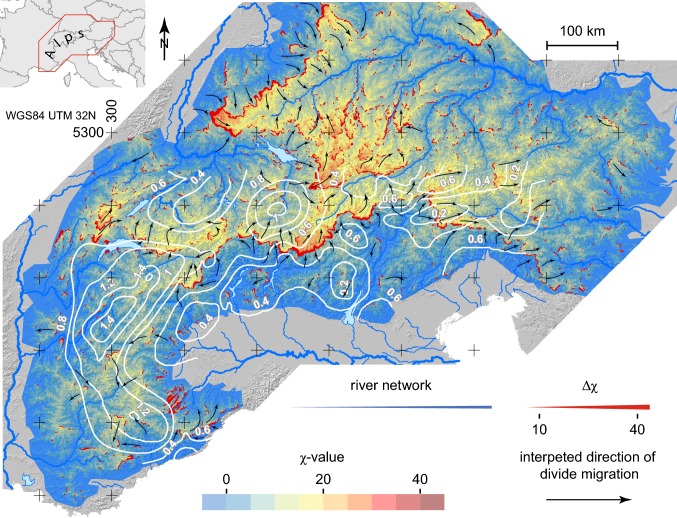
Interpretation of divide mobility, based on χ within individual catchments. Colour shading indicates average χ from channels (Fig. 2) in the local catchment area. The weight of the red line marking divides scales with the magnitude of Δχ between catchments. Black arrows indicate the inferred migration directions of the divides in order to reach geometric equilibrium (the length of the arrow does not indicate a rate). White contour lines show thermochronometric exhumation rates in mm per year for the last 2 Ma taken from Fox et al. (2015) and serve as a proxy for recent erosion rate

The χ-metric is a characterisation of the river channel network. However, much of its value is as an interpretive tool for the local channel catchment geometry. Therefore, we have presented the information on the χ-metric in a different format that emphasises the difference in the χ-values across major divide boundaries. To better visualise such differences at catchment boundaries, we display the χ-value from the local channel over the local catchment area i.e. what drains into this channel segment and is below the channel-forming threshold (Fig. 3).

To produce this χ-map, we approximated the catchment areas for an Alpine-scale analysis: We calculated χ-values from a raster grid, and averaged these values over 200 m increments to obtain a vector of the river network. The upper end of each network segment was used to calculate equal distance areas that cover the complete map extent (Delaunay 1934). ArcGIS was used for these tasks. This mimics the watersheds well on a map showing the whole Alps. Analysis with smaller catchments would require an exact spatial definition of the divide using a flow direction approach.

### Δχ across divides

We define Δχ as the difference in the χ-values from one catchment to another across a divide. The χ-values from the river segments on both sides are differenced at the watershed to calculate Δχ. The magnitude of Δχ can be interpreted as an indicator for divide instability, provided that uplift and rock erodibility are equal and that there are no major transients in river profiles (Willett et al. 2014). A finite value of Δχ implies migration towards the higher χ side. To visualise the χ-difference of the rivers we display Δχ with a red line at the divide (Fig. 3), with the line width reflecting the Δχ magnitude. To help with visualisation we do not display Δχ below 10.

### Minimum elevation (CRM) and catchment-restricted relief (CRR)

**Fig. 4:**
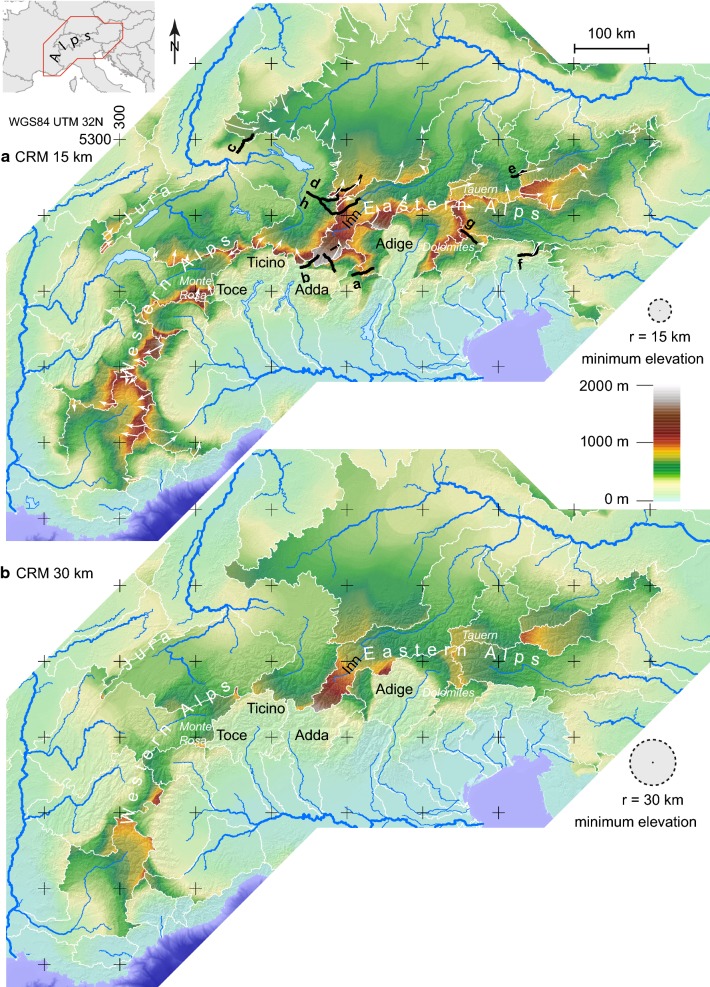
*Catchment restricted minimum elevation* (CRM) within a radius of 15 km (**a**) and 30 km (**b**) for present day main river catchments (see names of rivers on map in Online Resource 3). White lines outline catchments for the calculation. The location of the profiles from Fig. 5 are shown as black lines. Most high CRM values are north of the main Alpine divide

Relief and elevation distribution are well-established methods to analyse landscapes. Minimum and maximum elevation are important parameters of a landscape, and can provide complementary information to the χ-map, which contains no elevation information beyond what is needed to establish flow direction. The relief preserves the glacial history due to over deepening of valleys in the Alps where an ice cover was present. Typically, relief is defined as the difference between maximum and minimum elevation within a specified radius. Here, we have experimented with a variation of this method, by taking the elevation of any point in a catchment and comparing it to the minimum elevation within a specified radius. However, given our interest in catchment characteristics, we restricted the minimum elevation point to lie in the catchment of the point we examine, and refer to this quantity as *Catchment*-*Restricted Minimum Elevation* (CRM). This quantity is comparable to relief and river channel gradient (Fig. 4). CRM provides a basis to compare base level relief over divide boundaries and analyse the different ambient base levels of the rivers. The related quantity, *Catchment*-*Restricted Relief* (CRR) shows relief restricted to lie within a single basin (Online Resource 2). CRM and CRR together define the topography: *z*_*topography*_ = *z*_*CRR*_ + *z*_*CRM*_.

We used the digital elevation model and polygons from the European river catchments dataset (European Environment Agency) to separate the Alpine drainage system into different sub-catchments. We created a *Catchment*-*Restricted minimum elevation* (CRM) and a *Catchment*-*Restricted Relief* (CRR) map (Online Resource 2) using a circular moving window with a radius of 15 km and 30 km. To each point of the map, the minimum elevation (for CRM) or the relief difference within the basin and within that circle (for CRR) was assigned. The CRM provides the elevation of the local base level and CRR the remaining relief above the CRM. The assumed radius of influence of 15 km seems to meet the criterion of this erosion base because the map preserves areas of high elevation valleys. A comparison of the CRM and a conventional minimum relief map was also conducted (Online Resource 2).

### Manual analysis of the geomorphology

**Fig. 5:**
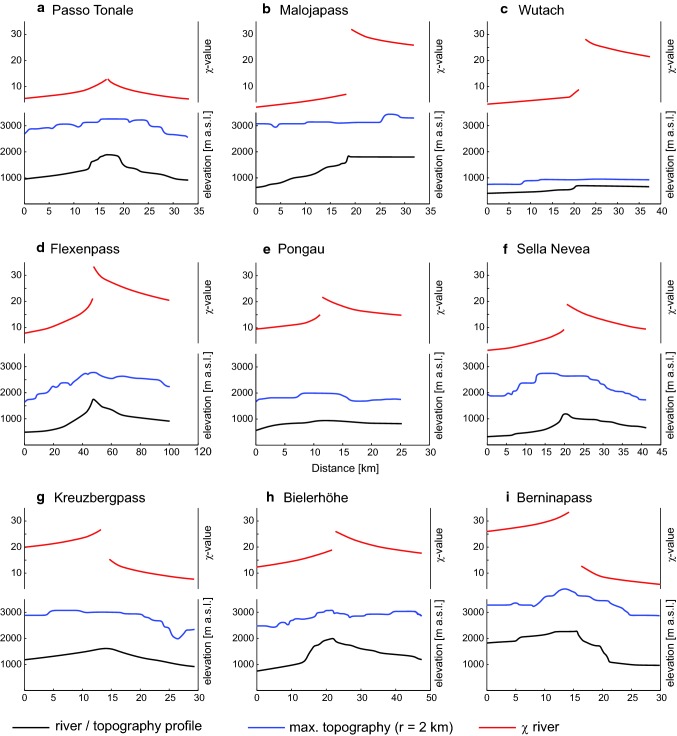
Elevation profiles along adjoining valleys and across drainage divides at major passes of the Alps. Black line indicates the elevation profile; blue line is the local (r = 2 km) maximum elevation; red is χ, note that the gradient of χ represents k_sn_. The profile locations are according to line and letter in Fig. 4. Asymmetry in elevation and χ reflect divide instability. Note that elevation and χ are independent and so provide independent evidence of divide instability. Profile **a** represents a divide that is in geometric equilibrium. Profile **b** shows the divide from the Inn to the Adda with an extreme asymmetry of the divide. Profile **c** is following the documented former course of the Wutach to the Danube prior to capture by the Rhine. Profile **d** is an asymmetric divide between the Rhine and Danube. Profile **e** represents a section along a major extrusion fault (see text). Profile **f** is an asymmetric divide over the main Alpine divide in the Eastern Alps. Profile **g** represents a symmetric divide with a Δχ that was probably introduced by a network reorganisation in the Pustertal. Profile **h** shows the asymmetry between the Inn and Rhine basins. Profile **i** is documenting the asymmetry and Δχ contrast around the Inn headwaters

As a complementary analysis, we manually studied the topography of the Alps with DEM maps and satellite imagery draped over topography (Google Earth). This is useful to verify the predictions of the χ-map regarding asymmetric divides. We present representative profiles for the topographical analysis in Fig. 5. Additionally, we identified low-relief watersheds and possible wind gaps in the Alpine region as a possible indicator of river transients (see marked wind gaps in Online Resource 3).

### Paleo-geography

**Fig. 6:**
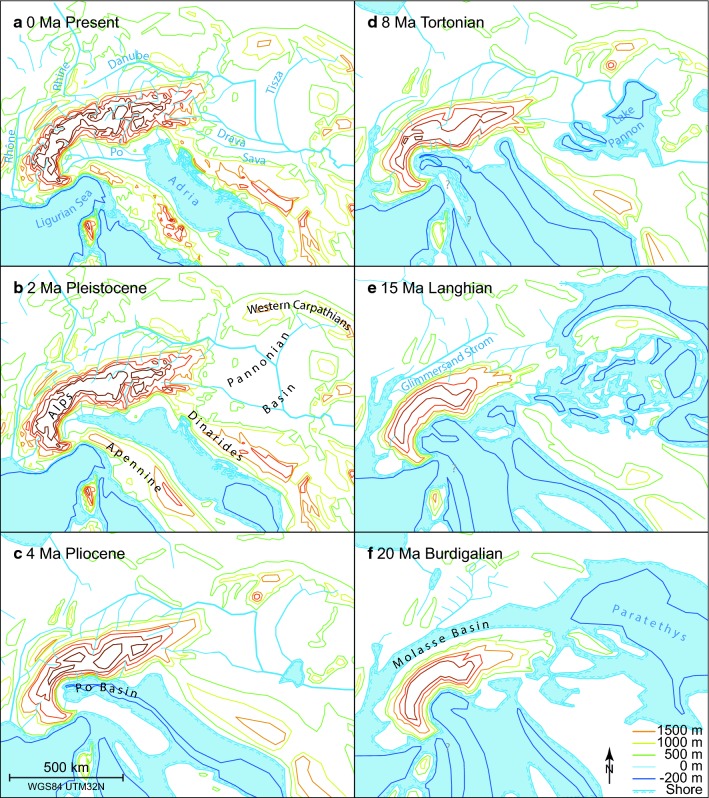
Paleo-geographic maps showing the Alpine and Pannonian geographic evolution (references in text). Note that the Danube formed as a longitudinal basin in the foreland of the Alps, and has subsequently elongated to the east following shoreline retreat of the Paratethys, which evolved into the modern Black Sea. The Rhine expanded mainly to the east

The stability of the basins depends on the geomorphic history of the river. Geological records offer a detailed archive for a description and interpretation of the geomorphic history (see Sect. 1.2). We constructed a set of paleo-geographic maps based on geologic observations from the literature (Fig. 6). We use geologic data such as provenance analysis and evidence of specific capture events, as well as information about paleo-river network location from incision and other available information to provide location and timing of events. The river networks and the elevation contours are primarily drawn after Kuhlemann (2007). The margins of Lake Pannon are based on Magyar et al. (2013). The paleo-geography of the northern foreland basin is taken from Kuhlemann and Kempf (2002). The Po Basin geometry and the Adriatic coast follow Boccaletti et al. (1990) and Ghielmi et al. (2010). The changes at the Rhine, Rhône and Danube junction follow Schlunegger and Mosar (2010). All maps are drawn in a fixed European reference frame that corrects the plate tectonic position of the Alps and Apennines with respect to Europe.

## Resulting maps

### χ-analysis

The χ-map exhibits high values in the headwaters of the Doubs and Aare River, in the Jura and the Alps of Western Switzerland (see Fig. 2 and detailed χ-map in Online Resource 3). A very distinct line swinging around Lake Constance separates areas with high values (warm colours) located in the headwaters of the Inn, Lech, Iller and Danube River from westerly adjacent areas with lower values, following the transition from the Western to the Eastern Alps. High χ-values are also found in the headwaters of the Salzach and Drava River further to the east. Very generally, the maximum χ-values in the Eastern Alps have a tendency to be higher compared with the Western Alps in France. Rivers that drain southward from the main Alpine divide have generally lower maximum values, even in their headwaters. In the western Southern Alps the χ-values are homogeneously low whereas in the eastern Southern Alps χ-values show more complexity and hence also some intermediate to high χ-values at channel heads abutting the major divides.

In the Western Alps drainage divides have a small Δχ (Fig. 3). Even over the main Alpine divide between the Rhône and the Po, there is only a small Δχ. Between the Alpine Rhône and the Po we see a moderate Δχ. The Δχ between the Rhine and Po is a little higher. Po and Inn rivers have the highest Δχ at the divide with the largest values being at the border with the Engadine Valley of the uppermost Inn. The Rhine and the Danube also share a high Δχ divide from the Alps to the Black Forest and the Swabian Alb north of the Molasse Basin. The Drava and Sava Rivers have a moderate Δχ against their southern counterparts. Within the Danube basin, the Lech and the Inn tributary both are large enough to also show a contrast in Δχ in the Alps.

The map also covers areas north of the Molasse Basin. We see that the high χ-values extend from the Eastern Alps into this region north of the Molasse Basin. The χ-contrast follows the divide from the Black Forest to the Bohemian Massif in the east. The high contrast in headwater χ is largely coincident with the catchment of the Danube.

### Catchment restricted topographic analysis

Catchment restricted minimum elevation (CRM) exhibits a number of distinctive features pointing to asymmetries over the main Alpine divide (Fig. 4): There is a zone of high CRM that follows the main Alpine divide starting in France in the Western Alps, continuing to the Monte Rosa Massif in the Central Alps. North of the Ticino River (see Online Resource 3), the CRM has lower values. The maximum value for CRM is located in the headwaters of the Inn, where values are high in the upper reaches, reflecting the fact that the Inn flows above 1000 m a.s.l. for more than 100 km north-eastward from Maloja Pass through the Engadine. Also, the catchment restricted relief (CRR) is low in the Engadine (Online Resource 2). Within the Eastern Alps, the area occupied by the Tauern Window is an area of high minimum topography, as well as the Dolomites area. The main water divide of the Alps appears as a zone of high CRM from the Western to the Eastern Alps. The CRM illuminates a stark difference at the catchment boundary of the Po to the west and in the headwaters of its northern tributaries (Toce, Ticino, Adda and Adige). All southern minima are significantly lower than the northern counterparts.

## Interpretation of the Alpine river basin structure

### Current state of river basins

Alpine rivers define large catchments and drain to three distinct marine water bodies. The geometries of the main river networks show first-order differences in the maximum χ-values (Fig. 3). This demonstrates distinct geometric differences amongst the main basins of the Danube, Rhine, Rhône and Po rivers. The catchments of the Danube river tributaries have the largest maximum values of χ. Significantly lower maximum values are seen for the Rhine, Rhône and Po catchments. These differences exist in spite of similar maximum relief in each catchment. The drainage basin structure and χ-values primarily reflect the disparity in the distance to base levels, which clearly differs amongst the basins; the Rhône and the Po drain close to the Alps into the Mediterranean whereas the Rhine drains to the North Sea and the Danube has some 1300 km to base level in the Black Sea.

Topographic analysis can test whether or not the river geometry is reflected in the geomorphology. The χ-map interpretation can be tested in that it makes a prediction where we find asymmetric divides. Nine representative longitudinal profiles over water divides show different geomorphic characters (Fig. 5). This is evident in both the elevation profile of the channel and the mean topography. The profile (Fig. 5a) at the Passo Tonale is a good example for a divide in equilibrium. Both sides of the divide are symmetric. Most other major passes in the Alps show some degree of disequilibrium, including the transient at the Danube water divide at the Malojapass, the Wutach, the Flexenpass and the Sella Nevea (Fig. 5b–d and f). All these divides show a difference in χ, a steeper channel profile towards the low χ-side and a flatter channel towards the high χ-side. Profiles **e** and **g** show only a small difference in slope on either side of the pass. The Pongau is located along a large strike slip fault (Salzach-Ennstal-Mariazell-Puchberg Fault). The situation at the Kreuzbergpass located close to the Periadriatic Fault is special in that the divide seems to have been shifted to a less stable configuration which drains towards the Drava. Some remarkable asymmetric divides are illustrated in Fig. 5: For example the Flexenpass represents a transient divide showing a spatial migration signal through its asymmetric erosion rate proxies. The Malojapass also has a very asymmetric divide. There is geomorphic evidence that the Inn formerly extended further to the west where it drained two more valleys (Val Maroz and Val Forno) before the rivers were presumably redirected and currently flow to the Po. On the southern watershed of the Engadine we find an almost continuous line of asymmetric divides over several mountain passes including the Bernina Pass. This is a strong result supporting our interpretation of the divide instability due to geometric disequilibrium as characterised by the χ-map.

Both wind gaps and documented river network changes provide direct evidence for river transients. Wind gaps denote abandoned river courses due to river network reorganisation. From the Black Forest to the Swabian Jura and the Bohemian Massive we find an escarpment with high Δχ and river courses with known river captures events (Strasser et al. 2009; Yanites et al. 2013). Dry valleys from abandoned rivers in the Swabian Alb and wind gaps are frequent (Rutte 1987; Strasser et al. 2009). The Wutach capture at Blumberg is a well-documented example (Einsele and Ricken 1995). Also, the river geometry of the Drava and the Mur have high Δχ contrasts in the headwaters, both rivers converge with the Danube in the Pannonian plain, distal to the Alpine uplifts. This points to a common cause for high Δχ-values in the Danube catchment area that is located outside of the Alps. All wind gaps have a flatter profile towards the Danube system and a steeper one towards the river that took over the catchment (Fig. 5). The entire Danube catchment is prone to river network transients. This is also underpinned by the cumulative occurrence of river transients (e.g. wind gaps) in the Danube basin. The χ-map and the high Δχ line predict the occurrences of most of the wind gaps (see on χ-map in Online Resource 3).

Another type of high Δχ divide is found in the foreland fans where fan deposition results in a distributive channel network that does not follow the usual slope-area scaling (Willett et al. 2018). These fans are found in little-eroded basin parts such as the western Po or eastern Molasse Basin (see for example the bend of the Mangfall River west of Rosenheim in Online Resource 3).

Another metric for asymmetry is provided by the catchment restricted minimum elevation (CRM) map. Few catchment headwaters have a CRM above 1000 m (Fig. 4). The Rhône and its eastern headwaters Isère and Durance in the Western Alps, as well as the Danube tributaries in the Eastern Alps, the Drava, Mur and the Inn have high CRM values. Areas with particularly high CRM values are located in the upper Danube catchments (particularly the Inn). Large parts the headwaters of the Inn and its tributaries have CRM of more than 1500 m (Fig. 4). Interestingly, the maximum CRM along the crest of the Alps differs little from west to east. In contrast to χ the CRM is a local quantity, restricted to the 15 km radius used for its calculation. In spite of this shorter length scale, we can interpret the CRM in terms of basin stability in order to compare it with χ. A steady state divide would have similar elevation distribution on either sides of the divide and therefore identical CRM. Unequal minima would lead to a catchment expansion in favour of the lower CRM elevation because of a lower local base level within the same radius. We can then directly compare CRM with the χ-map and differentiate local instabilities from basin wide geometric instabilities. Comparison with the mapped river transients reveals that the χ-metric predicts transients better than the relief metrics. We suspect that the glacial overprint of the topography affected CRM much more than χ. The valleys of the Alps were deepened during glaciation but the position of the divides largely remained fixed, so relief metrics are more likely to be controlled by local effects, rather than large-scale geometric instabilities.

### χ-sensitivity to steepness and base level

The drainage network of an orogen includes high-relief regions characterised by bedrock rivers and flat, low-relief alluvial areas beyond the mountain front. A typical river in the χ-analysis has bedrock reaches with high steepness and alluvial basin reaches with a lower steepness. The alluvial reaches of most major rivers are likely to be sediment transport-limited and have little or no tectonic uplift, which implies a lower steepness for these alluvial rivers. The transition to the alluvial reach is expected to be at or near the mountain front where we also see a change in uplift and exhumation rates. The transition elevation splits the total river relief of a river basin in an alluvial reach relief (ARR) and bedrock reach relief (BRR). However, the transition occurs at different elevations for every river, so the ARR and the BRR generally vary from basin to basin and it is important to assess potential errors from these differences.

A χ-calculation integrates catchment area from a base level, assuming a slope area relation. In steady state, χ can be interpreted as a proxy for elevation and much of our interpretations of divide mobility is based on this. Although, in a steady system with distinct bedrock and alluvial reaches, the predicted elevations diverge from a model that assumes uniform steepness within the analysed area.

Sea level is the obvious choice for base level when performing a χ-calculation. However, we introduce an error in predicting the χ-values because we include the alluvial reaches of rivers. Ignoring the alluvial reach steepness, we assume bedrock steepness for the contribution of χ in the alluvial plain. The simplification results in an overestimation of the χ-values (Forte and Whipple 2018).

**Fig. 7:**
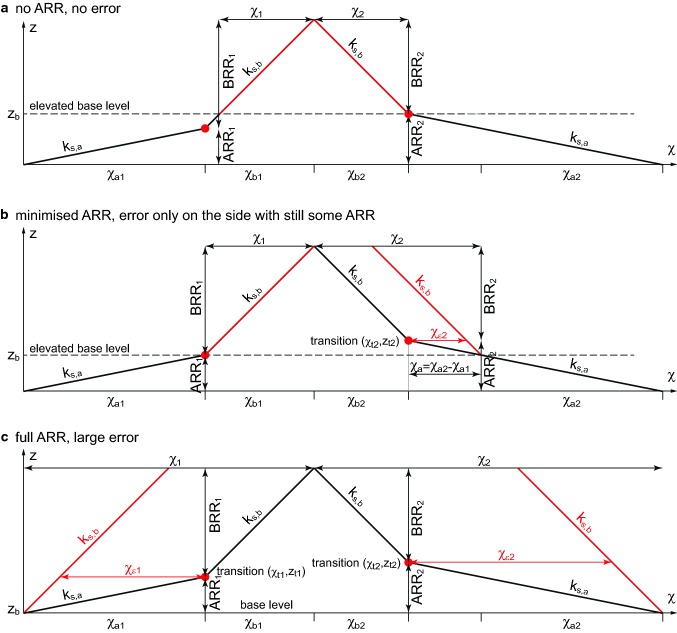
Error assessment over a divide due to a change in river steepness that is not included in the reginal χ-calculation. Analysis assumes that there is a low steepness alluvial reach and a higher steepness bedrock reach. The bedrock reach relief (BRR) and the alluvial reach relief (ARR) define the total river relief. Red is the calculated χ-plot for a standard analysis with conventional definition of χ. Black shows the true χ-plot. The change in steepness is marked as transition with a red dot. **a** Shows the result for the case where base level is set at the maximum ARR, z_b_ = ARR_max_, **b** shows the case where the base level is at the minimum ARR, z_b_ = ARR_min_, and **c** assumes base level at nominal sea level, z_b_ = 0. χ_1_ and χ_2_ represent the inferred value of χ on either side of the divide, whereas χ_a1_, χ_a2_ and χ_b2_, χ_b2_ represent the χ-values for the alluvial reach and bedrock reach, respectively. The error in the analysis, χ_ε_ is given by the difference between the red and black χ-profile

In this section, we conduct a sensitivity analysis by assuming that there are two distinct steepnesses, a lower steepness in the alluvial reach near base level, *k*_*s*,*b*_, and a higher steepness for the bedrock reach, *k*_*s*,*a*_. In this simple case, we predict the elevation from two sides of a divide that have different transition elevations. Assuming equilibrium at steady state, the profiles should reach the same elevation at the divide. The difference between the two predictions from the calculation is the error *z*_ɛ_. Calculating the elevation error also allows us to estimate the error in the χ-quantity, *χ*_*ɛ*_, as the two are related through the steepness (see Fig. 7). The equation for the elevation at the divide can be separated into a term representing the χ-calculation and a second being the elevation error *z*_*ɛ*_ (square brackets).

$$k_{s,b} \chi_{b1} = k_{s,b} \left( {\chi_{b2} + \chi_{a2} - \chi_{a1} } \right) - \left[ {\left( {k_{s,b} - k_{s,a} } \right)\left( {\chi_{a2} - \chi_{a1} } \right)} \right]$$

$$z_{\varepsilon } = \left( {\chi_{a2} - \chi_{a1} } \right)\left( {k_{s,b} - k_{s,a} } \right)$$

We consider first the case where we take the base level to be at the lowest transition elevation. With this elevated base level we have *χ*_*a*1_, = 0 and we can redefine *χ*_*a*2_ − *χ*_*a*1_ = *χ*_*a*_ as the remaining alluvial reach in the analysis. The transition elevation of the remaining alluvial reach is *z*_*t*_. The mismatch is the error, also read as *χ*_*ɛ*_.

$$z_{\varepsilon } = \chi_{a} \left( {k_{s,b} - k_{s,a} } \right)$$

$$\chi_{\varepsilon } = \frac{{z_{t} }}{{\left( {k_{s,b} - k_{s,a} } \right)}}$$

**Fig. 8:**
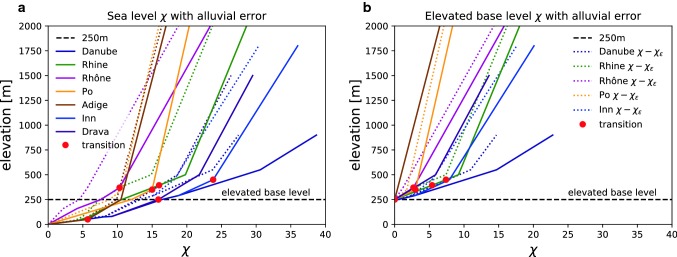
χ-profiles and χ_*ɛ*_ error with different assumed base levels. Error is estimated for the case of a low steepness alluvial reach, not included in the standard analysis. The two steepnesses for the error assessment are k_s,bedrock_ = 100 and k_s,alluvial_ = 20. The alluvial steepness is lower therefore the error is always negative. The error is smaller with the elevated base level. The horizontal shift between the figures is described in Online Resource 1

The error in the analysis has contributions from each steepness and is therefore dependent on the steepness contrast and the elevation of the transition. Under the premise of two distinct steepnesses, higher base level will minimise error (Fig. 8). If we include more ARR the error becomes larger. Conversely, there is no error in an upstream χ-integration if the base level is higher than all ARR. Theoretically a high base level is thus preferable to remove the error due to a lower alluvial reach.

However, a higher base level introduces other complications with a χ-analysis. With a high base level, large regions are removed and therefore catchment area potentially out of equilibrium is not included in the analysis. Consequently, with a higher base level, the χ-analysis risks that the base level is in a transient state (e.g. incising), which violates the condition of this point for cross-basin comparison.

An elevated common base level that still includes alluvial plains is a reasonable compromise. We use an elevated base level of 250 m that cuts away most areas with mainly alluvial basins (see Online Resource 1 Fig. 3). The two remaining alluvial reaches in our analysis are parts of the Rhine and Danube Rivers. The Rhine enters the Alpine system in Basel at 250 m where it hits the Jura Mountains. The upstream areas are generally incising into the Molasse Basin, except for the over-deepened glacial valleys. The main tributary of the Danube, the Inn River, displays a similar pattern to the Rhine, while the Danube itself never reaches the Alps.

The situation around the Alps is complicated by glaciation, which shifted the bedrock alluvial transition. The steepness change at the mountain front is also rarely distinct because of the deposition of glacial sediment in over-deepened valleys. However, our estimated errors of the Alpine χ-analysis are everywhere much smaller than cross-basin differences at the drainage divides, so we expect that our analysis is robust with respect to the base level question (see Online Resource 1), a conclusion confirmed by the correlation with asymmetric divides as discussed below.

### Paleo-geographic constraints

We compiled paleo-geographic maps to illuminate the temporal variability of the river network for an interpretation. The χ-analysis provides information on the river network geometry for the present day, which is interpreted in terms of network changes and dynamics. Therefore, the maps are also interpreted in terms of past events. We discuss here the important reorganisations of the river networks north of the Alps in the NAFB as well as the evolution of the main basins to reconstruct the geomorphic history of the whole domain (Fig. 6) and reinterpret the reasons for divide migrations.

After a first marine regression in the Oligocene, the deposition of the Lower Freshwater Molasse was associated with east-directed transport in the northern Alpine foreland basin (Kuhlemann and Kempf 2002 and references therein). Following the Burdigalian the marine transgression of the Upper Marine Molasse (Fig. 6f) and during deposition of the Upper Freshwater Molasse, sediment transport was directed to the west in northern Bavaria (Fig. 6e). Evidence for this includes the late Burdigalian Graupensand sediments that filled an incised valley before the Serravallian (Buchner 1998). The Graupensand- or Glimmersand River drained the Molasse basin and surrounding northern areas, including the Bohemian massive, flowing towards the west until about 10 Ma (Ziegler and Fraefel 2009). It is unknown if Alpine rivers also fed into this river system or were part of an independent river system possibly flowing to the east. Subsequently after 11 Ma, the thrusting of the Jura blocked this entire system and reversed the drainage direction to the east where it formed the Aare-Danube system (Fig. 6d). With the reactivation of the Bresse Graben, the Aare River established a path circumventing the Jura to the north (Fig. 6c). This path is well documented in the 4.2–2.9 Ma old Sundgau gravels (Giamboni et al. 2004). This new west-draining system re-established the westward drainage direction in front of the Jura, rerouting the Aare back to the Rhône Graben. It is not clear if the Alpine Rhône drained into the Aare at this time or if the Rhône already had its current path (Villinger 2003). The capture of the Aare-Danube triggered a massive sediment pulse that may have obstructed the Aare-Doubs in the Sundgau. At the time the Sundgau gravels were deposited, a line from the Säntis to the Black Forrest marked the continental divide between Rhône and Danube, the Alpine Rhine drained towards the Danube (location see Online Resource 3).

After 2.9 Ma, infill of heavy minerals with Alpine origin document the rerouting of the Aare into the Rhine Graben (Ziegler and Fraefel 2009 and references therein) (Fig. 6b). The base of the Rhine Graben was about the same elevation as the Bresse Graben (Villinger 2003). Therefore, the authors explain the rerouting with a shorter path to the ocean (Villinger 2003), reactivation of the Rhine Graben (Ziegler and Fraefel 2009) or transpression and folding in the Rhine Bresse transform zone (Giamboni et al. 2004). The activity of the Rhine Graben resulted in uplift of the horst shoulders of the Vosges, the Black Forest and since the Pliocene also the Sundgau region, although the main graben (Upper Rhine valley) continued to subside (Schumacher 2002). The short-lived path through the Bresse Graben could also be a late effect of the Messinian sea level drop that caused significant incision in the Rhône Valley up to the Bresse Graben (Clauzon 1982). We speculate that the subsequent filling of the incised Rhône valley after the Messinian degraded the advantageous profile, which allowed the deposition of the Sundgau gravels and possibly enabled the Rhine to capture the Aare-Doubs.

Further subsidence in the upper Rhine Graben (Ziegler and Fraefel 2009) and glacial perturbations (Villinger 2003) are explanations for the capture of the Danube-Rhine above todays Lake Constance at around 1.7 Ma (Fig. 6a). Documented captures by the Wutach near Blumberg ca. 20 ka ago show that the expansion of the Rhine basin was mainly at the cost of the Danube catchment (Einsele and Ricken 1995; Ziegler and Fraefel 2009; Yanites et al. 2013). This asymmetric growth of the Rhine mainly to the east towards the Danube and only little to the west suggests that the Danube was more vulnerable to capture.

The Pannonian Carpathian region east of the Alps has a dynamic geomorphic history (Fig. 6). The subduction of the European plate below the Carpathians triggering the opening of the Pannonian Basin must have affected the Danube and all other rivers draining to the Pannonian. Shortening in the Carpatho-Balkan orogen created a barrier (breached at the Iron Gate) and caused a water level raise after 12.6 Ma in an expanding Lake Pannon (Central Paratethys) (Palcu et al. 2015; Kováč et al. 2018). The base level defined by Lake Pannon evolved independently from global sea level, and is therefore difficult to establish. Bio-stratigraphic markers document a connection to Lake Pannon from the Eastern Paratethys at 6.1 Ma (Popov et al. 2006). At present, the outlet of the Pannonian Basin is at 100 m a.s.l. The regression of the Eastern Paratethys (the Black Sea) elongated the Danube basin significantly (Popov et al. 2006). This change of the river geometry triggered a cascade of profile adjustments still observable in the present day river basins and χ-maps. The disadvantageous geometry potentially facilitated catchment loss to the Rhine and other neighbouring basins.

Indentation and extrusion tectonics are the dominant processes in the Eastern Alps. Rather than growth by simple frontal accretion, much of the shortening in the Eastern Alps has been taken up by strike-slip faults that have allowed lateral extrusion of material to the east. Most of the main rivers in the Eastern Alps follow these fault structures (Frisch et al. 1998). The Pongau section (Fig. 5) represents such a fault-parallel valley at a location where the river leaves the trace of the fault.

There may be a remaining influence on the morphology by the base level change associated with the Messinian salinity crisis (Robl et al. 2017). Sea level was lowered by up to 2.5 km some 5.5 Ma ago. During this stage, deep canyons were formed below today’s Po Basin (Bini et al. 1978) and Rhône valley (Clauzon 1982). Today, lakes south of the Alps have their deepest points below sea level (Bini et al. 1978). Some of these pre-existing deep valleys were possibly widened and deepened during glaciations (Bini et al. 1978). These intra-montane basins shifted the influence of the Po Basin northward especially where the over-deepened valleys reached far into the Alps (e.g. Adige or Ticino). Presumably in late Miocene the NAFB was uplifted to 400–500 m above sea level, whereas the Po Basin on the southern side remained close to sea level. The Messinian sea level drop caused a strong erosion signal on the southern side, and models suggest that this drop may have caused a shift of the main Alpine divide (Robl et al. 2017). The inherited asymmetry would also cause a Δχ signal at the main Alpine divide as seen in the χ-map. This model fits with the interpretation that the Eastern Alps have a stronger migration tendency than the Western Alps.

### The Danube as a continental-scale victim basin

The Danube has a history of losing catchment area due to a disadvantageous basin geometry. The Pliocene migration of the mouth of the Danube towards the east has lengthened the path that water and sediment must travel, lowering the average gradient and sediment transport capacity. This is reflected in the current high values of χ in the upper catchments of the modern Danube. The reason for the stark contrast with its neighbouring basins is that they have much shorter paths to traverse, but the same total relief, and so must have higher average steepness. The river geometry is weakening the erosive power of the Danube. Low erosive power makes the Danube more susceptible to uplift, and thus more vulnerable to area loss to surrounding basins including the Rhine Basin.

Presently, the Danube catchment is losing area from all sides, but most recognisably to the Rhine. The Rhine offers a shorter path to sea level and dropped the gradient towards the north. This gradient was steeper than the one towards the Doubs or Rhône respectively that first captured the Aare from the Danube, leading to the series of capture events as described above (Einsele and Ricken 1995; Yanites et al. 2013).

The Paleo-Danube drained into the Central Paratethys, which became Lake Pannon after its isolation from the Eastern Paratethys by the Carpathians around 12.6 Ma (Palcu et al. 2015). After isolation the water level increased, and the sediments from the Danube progressively filled the Lake from the northwest to the southeast forming a large alluvial fan, until the Pannonian Basin was completely filled by about 4 Ma (Magyar et al. 2013). The course of the Danube became elongated to the Black Sea in the Pliocene; a new base level was established. The migration of the delta through Lake Pannon itself did not reduce the erosional power of the upstream river. However, the uplift of the basin from sea level to its present elevation of about 100 m above sea level slightly lowered the river gradient above.

The evolution of the Carpathian Pannonian region had consequences for the complete Danube basin. The erosional capacity dropped, triggering a response upstream. The pre-existing disadvantage of the Danube was amplified after the Rhine started draining the Molasse Basin. Starting around 4 Ma, the Danube headwaters in the western Molasse basin were captured by both the Rhône and Rhine. (Kuhlemann and Rahn 2013). The catchment area that today drains the Alpine Rhine into Lake Constance was captured from the Danube in the Early Pleistocene. This capture caused a pulse of incision in the Rhine drainage network that is still advancing the water divide into Danube catchments (Yanites et al. 2013). With the Rhine entering the Alpine system and the uplift of the Swiss Molasse Basin, efficient erosion started (Villinger 2003; Ziegler and Fraefel 2009). The Eastern Molasse Basin remained in the Danube Basin and therefore was not as strongly affected by erosion. Lower gradients of river profiles in the basin have an effect on the regional erosional potential that facilitated and probably triggered the change of the drainage direction towards the Rhine. Following capture, fluvial incision was rapid and led to erosion and removal of sediment in the Lake Constance headwaters. Strikingly, the Alpine Rhine catchment exhibits high rates of exhumation over the last two million years as discussed in the next section (Fox et al. 2016) (Fig. 3).

The analysis presented here suggests that it is the geometry of the Danube drainage basin, including its long path to base level, which is driving the net reduction of the drainage area. As such, it is likely that the Danube has a long history of area loss and inward motion of the water divides. The recent, documented captures are only the latest stages of this protracted history. The χ-analysis reveals an explanation why most recognised reorganisation events are documented along the margins of the Danube basin.

We see that the cause for loss of headwater area lies within the Danube catchment. In addition, we observe that the Rhine mainly expanded to the east of the Rhine Graben and not much to the west (e.g. Cordier et al. 2004). All this is implying an additional cause and effect relation independent of the Rhine. Assuming that the driver of major drainage reorganisation is the Danube basin geometry, then the cause must lie in the early river paleo-geography that established the Danube as an orogen-parallel, longitudinal river, thus establishing its susceptibility to capture from surrounding rivers. The Danube river network geometry facilitated river capture to the Rhine.

### Implications for long-term erosion patterns of the Alps

The exhumation pattern of an orogen is mostly dependent on its tectonic evolution. However, exhumation requires erosion of rock and removal of mass out of the orogen. Rivers are an important agent in both eroding rock and transporting sediment. The river channel gradient is linked to the erosion rate and its potential to transport mass over long distances. Changes in river gradients affect the whole basin, although time is required to propagate the gradient change through the river network. Such a change can be a higher river gradient that causes higher erosion and higher exhumation in a basin. This relation between exhumation and river gradients is observable throughout the Alps.

Local erosion rates over a drainage divide correlate directly with the divide migration rate (Whipple et al. 2017). Erosion rates experience the greatest change if a river capture shifts the course of a river and increases the gradient regionally.

The Alpine exhumation model of Fox et al. (2016) shows spatial and temporal patterns of exhumation, explained mainly with tectonic and climatic drivers. The model shows anomalously high exhumation rates over the last two million years in the Western Alps and the Alpine Rhine area above Lake Constance. Subduction-related slab tectonics and glacial erosion have driven exhumation in the Western Alps (Fox et al. 2016); but there is no obvious tectonic or climatic cause for the high exhumation rates in the Alpine Rhine area. In this area, we see a χ-contrast between the Alpine Rhine and Danube tributaries, the location of low χ-values coincides with the zone of anomalously high exhumation (see Fig. 3). Given the dynamic changes in the downstream river basins outside the Alps, we suggest the Rhine had multiple, progressive capture events and cannibalised drainage area from the Danube, in particular the former Alpine Danube. Therefore, the high exhumation rate might be related to the transfer of drainage area from the Danube to the Rhine. A capture drops the base level for the captured reach, and creates a steeper river gradient that supports efficient transport and enhanced exhumation. This major network change outside the orogen is likely to be visible in the orogen as a change in exhumation rates in the respective river basin. Hence, Alpine exhumation co-evolved not only with the tectonic evolution and climate, but also with river network perturbations in the entire river basin.

Other studies suggest that the exhumation peak in the Alpine Rhine is related to mechanically weak rocks that caused landslides (Korup and Schlunegger 2009). Weak rocks facilitate regional isostatic rock uplift and enhances fast erosion, but exhumation still needs an efficient transport mechanism that exports the mass out of the Alps. The change of drainage direction in the Alpine Rhine from the Danube to the Rhine probably increased erosion and transport rates and would thus be a component of lithologic-driven erosion enhancment.

The main Alpine river basins connect distinct tectonic domains, where a regional tectonic perturbation (e.g. uplift) will influence the entire river basin, regardless of its position within or outside the Alps. Consequently, erosion rates will also respond to this perturbation. In summary, a comprehensive interpretation of the exhumation pattern in the Alps must also include changes in the river basins outside the Alps.

## Summary and conclusions

Two independent surface analyses were conducted: River geometry analysis with the χ-method and elevation distribution analysis with *catchment restricted minimum elevation* (CRM) and *catchment restricted relief* (CRR) metrics. The calculations were done for the greater Alpine region and interpretations considered the full extent of the Alpine river basins.

Basin geometry analysed with the χ-metric shows systematic differences in river geometry. The main Alpine river basins (Rhine, Rhône, Danube and Po) have a distinct geometric character; the Danube basin boundaries are prominently visible in the χ-map due to the strong contrast in maximum χ-values at the watersheds. The main Alpine divide is reflected in Δχ-values and is even more prominent in CRM differences over the divide. We conclude that Alpine rivers are not in geometric equilibrium. Hence, the observed river transients to restore equilibrium are a logical consequence.

The relationship between river basin stability across watersheds and Δχ-values is confirmed by river profiles, which show corresponding differences between channel morphology and Δχ. For example, the Danube River has anomalously low steepness and high χ in its upper reaches. We observe a consistent polarity of asymmetry for both plan-form geometry (characterised by χ) and vertical-section profiles, suggesting that the basin geometry is responsible for the disequilibrium and water-divide migrations.

Contrary to previous interpretations, the expansion of the Rhine is not exclusively attributed to the tectonic evolution of the Rhine Graben; the disadvantageous shape of the Danube basin has also contributed to the changes in drainage pattern. It is noteworthy that the Rhine expanded mainly to the east at the expense of the Danube basin. The lower gradient of the Danube is a result of its original longitudinal basin form, exacerbated by the uplift of the Carpathians and subsequent basin elongation. The river network propagated the reach of influence of this profile change in the Carpathian region to the complete Danube basin. Hence, the river captures of the Rhine are promoted by tectonic events well outside the Alpine orogen and the Rhine Graben system. River captures of the Rhine from the Danube removed discharge that further weakened the erosive potential of the Danube River. Hence, transients and river captures to the disadvantage of the Danube catchment are likely to continue in the long-term future.

The geomorphic history demonstrates that the river network geometry was set by Alpine tectonics and geographic boundary conditions, but that this system has been in geometric disequilibrium since the onset of orogeny. Following convergent tectonics, the disequilibrium of the river system remained, the changes in drainage basin geometry are a consequence of this inherited disequilibrium. The results demonstrate that the geometric shape of drainage basins, including the regions outside the immediate Alpine area, have a significant influence on Alpine geomorphology and erosional processes.

## Electronic supplementary material

Below is the link to the electronic supplementary material.

Supplementary material 1 (PDF 2022 kb)

Supplementary material 2 (PDF 4412 kb)

Supplementary material 3 (PDF 9597 kb)
