# Supplementary material for: Greater Alpine river network evolution, interpretations based on novel drainage analysis
Source: Swiss J Geosci. 2018 Nov 22;112(1):3–22. doi: 10.1007/s00015-018-0332-5 (PMC7081830; doi:10.1007/s00015-018-0332-5)
Supplement: Supplementary file 1 — Supplementary material 1 (PDF 2022 kb) [file 15_2018_332_MOESM1_ESM.pdf]

Error assessment of χ-values

Predicting the elevation at the divide can be separated in a term representing the χ-calculation with the term in the squared brackets being the elevation error z_ε (see Fig. 7).

$$BRR_1 + ARR_1 = BRR_2 + ARR_2$$

$$k_{s,b}\chi_{b1} = k_{s,b}(\chi_{b2} + \chi_{a2} - \chi_{a1}) - \left[(k_{s,b} - k_{s,a})(\chi_{a2} - \chi_{a1})\right]$$

$$z_\varepsilon = (\chi_{a2} - \chi_{a1})(k_{s,b} - k_{s,a})$$

With elevated base level we have $\chi_{a1}, = 0$ and we can redefine $\chi_{a2} - \chi_{a1} = \chi_a$ as the remaining alluvial reach in the analysis (see Fig. 7). The mismatch is the error. The error can also be read as an χ-error, χ_ε.

$$z_\varepsilon = \chi_a(k_{s,b} - k_{s,a})$$

$$\chi_\varepsilon = \frac{z_t}{(k_{s,b} - k_{s,a})}$$

The χ-values in this study have a base level of 250 m above sea level. To compare the χ-values from this study with the base level of 0 m a.s.l. we list here χ_d . The χ_d-quantity is taken from Giachetta and Willett (2018).

$$\chi_d = \int_{x_b=0}^{x=250} \left(\frac{A_0}{A(x)}\right)^{\frac{m}{n}} dx$$

ESM 1 Table 1: Elevation of the Alpine front, maximum χ_{max} of the catchments and assessed error χ_ε at the transition (negative χ_ε values refer to an overestimation of χ and negative to an underestimation). The χ_d-quantity for the large rivers and indication of a nearby location to the 250 m a.s.l. line.

River	$z_{mountain\ front}$	$\chi_{\varepsilon,trans.}$	χ_{max}	χ_d	250 m a.s.l. location (nearby city)
Rhine	400	-1.9	29.8	10.6	Basel
Aare	550	-3.8	35.1	10.6	Basel
Neckar	none	none	30.6	13.6	Esslingen
Danube	none	none	38.7	16.4	Linz
Inn	450	-2.5	36.0	16.4	Linz
Traun	424	-2.2	28.2	16.4	Linz
Enns	300	-0.6	27.3	16.5	Enns
Drava	250	0.0	29.5	15.9	Maribor
Mur	350	-1.3	29.8	16.6	Leibnitz
Rhône	370	-1.5	41.7	7.6	Bellegarde-sur-Valserine
Doubs	none	none	35.9	13.4	Deluz (Besancon)
Isère	190	0.8	21.5	6.9	Montmélian (Chambéry)
Durance	80	2.1	25.2	3.7	Manosque (Aix-en-Provence)
Po	350	-1.3	21.0	12.0	Saluzzo
Dora Riparia	300	-0.6	20.8	10.0	Collegno (Torino)
Tanaro	600	-4.4	22.1	12.5	Fossano (Cuneo)
Ticino	193	0.7	19.1	12.6	Bellinzona
Toce	193	0.7	16.1	12.3	Domodossola
Adda	199	0.6	30.2	11.6	Sondrio
Adige / Etsch	50	2.5	17.0	10.5	Meran
Eisack	50	2.5	20.0	9.6	Bozen

Error assessment of χ-values

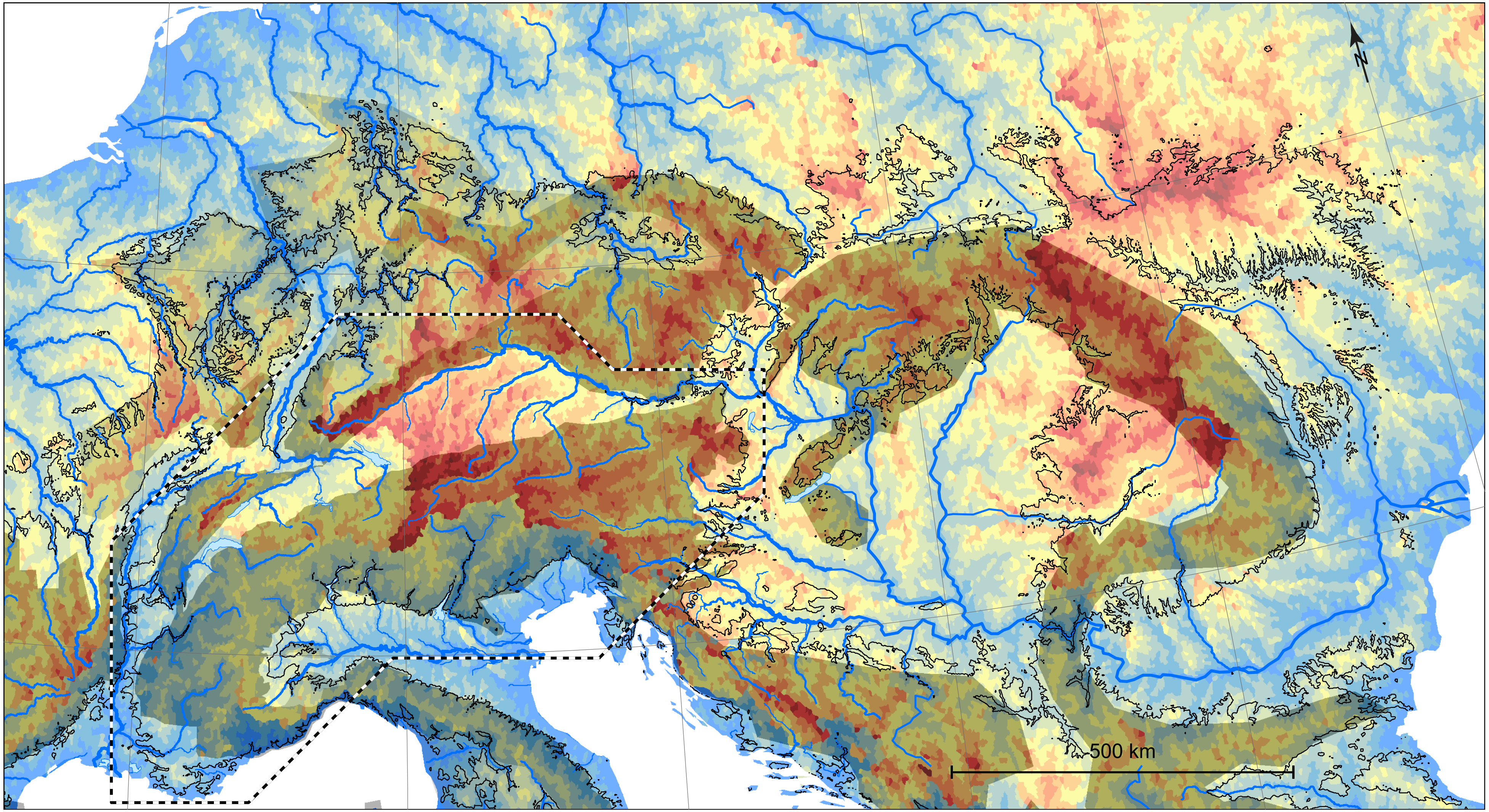

ESM 1 Fig. 1: Base level at sea level
European χ-map modified after Giachetta and Willett (2018). On this map sea level is the the base level of the χ-integration. The shading refers to an estimate of rock erodibilty (K) which shows where the alluvial basins are.
The map is projected on UTM 32N and has a 5° geographic grid.

250 m contour
outline χ-map
main river

K
10^{-6} 10^{-7}
weak basin — strong bedrock

χ-value (z_b=0 m)
0 20 40 60
