# Supplementary material for: Greater Alpine river network evolution, interpretations based on novel drainage analysis
Source: Swiss J Geosci. 2018 Nov 22;112(1):3–22. doi: 10.1007/s00015-018-0332-5 (PMC7081830; doi:10.1007/s00015-018-0332-5)
Supplement: Supplementary file 2 — Supplementary material 2 (PDF 4412 kb) [file 15_2018_332_MOESM2_ESM.pdf]

Relief analysis

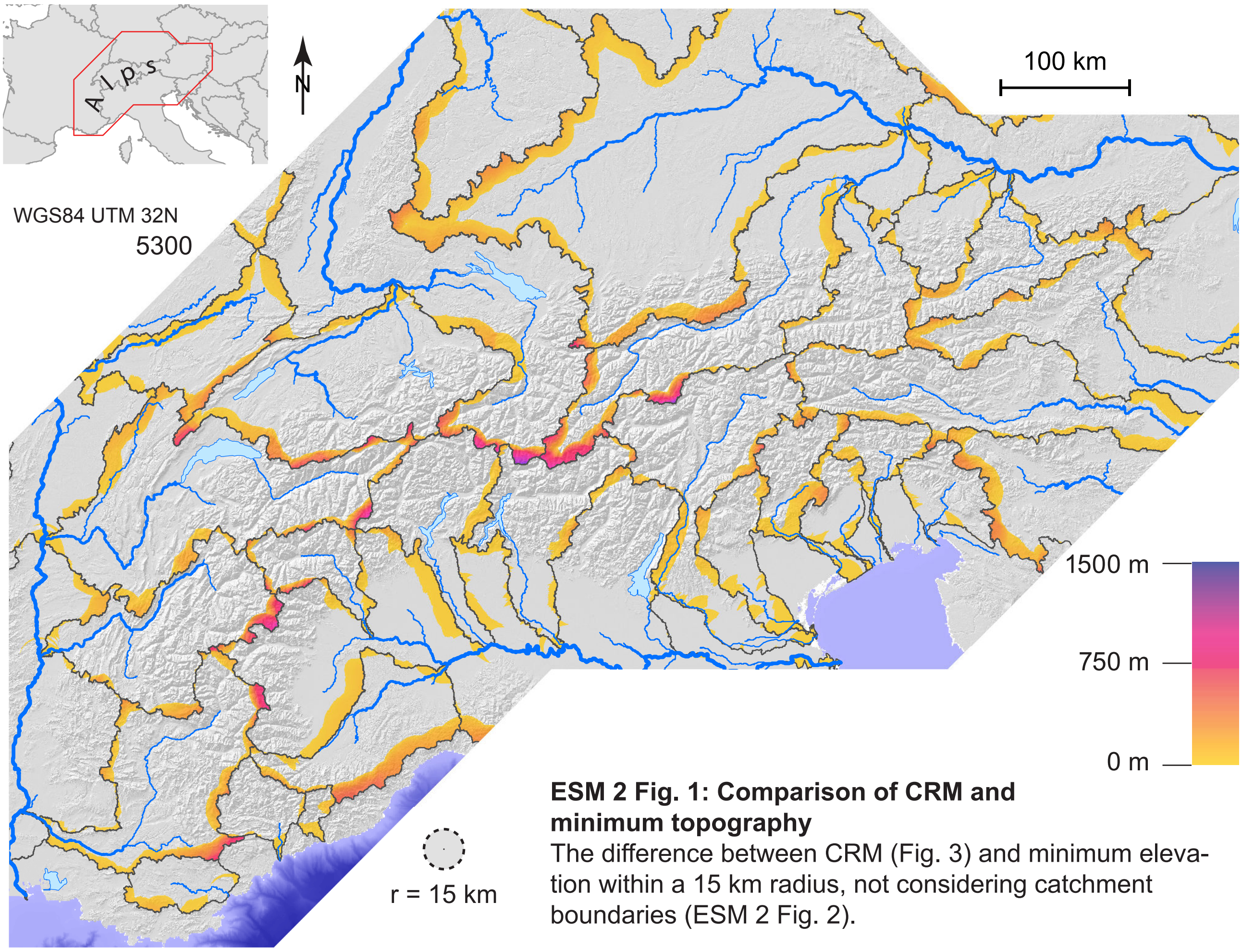

ESM 2 Fig. 1: Comparison of CRM and minimum topography
The difference between CRM (Fig. 3) and minimum elevation within a 15 km radius, not considering catchment boundaries (ESM 2 Fig. 2).

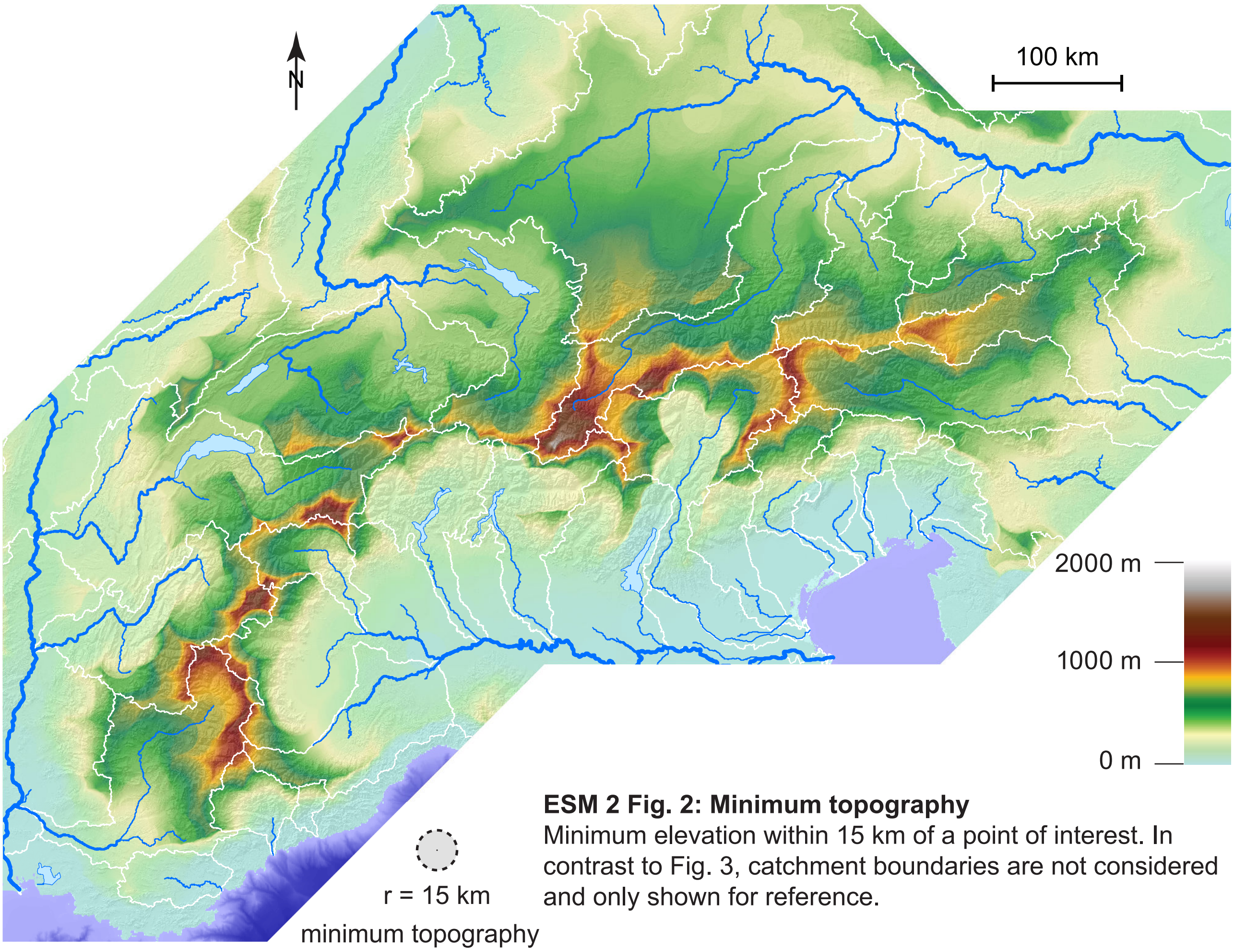

ESM 2 Fig. 2: Minimum topography
Minimum elevation within 15 km of a point of interest. In contrast to Fig. 3, catchment boundaries are not considered and only shown for reference.

Relief analysis

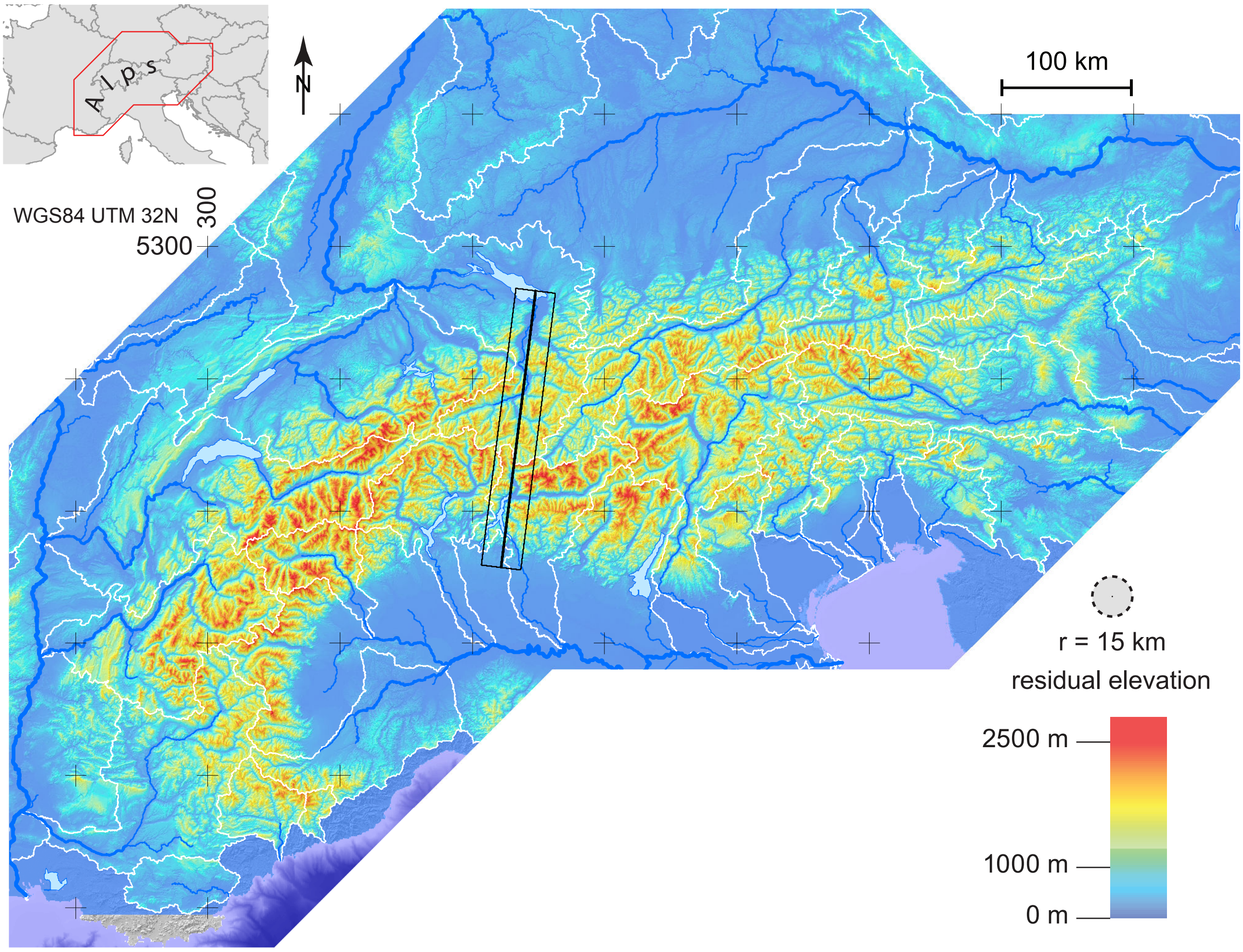

ESM 2 Fig. 3: *Catchment Restricted Relief* (CRR)
CRR with a radius of 15 km (see names of rivers in ESM 3). This is showing the topography only within the catchments. White outlines delaminate catchments used for the CRR calculation. The CRR is a measure for the local relief. Black line is the profile of ESM Fig. 4.
The headwaters of the Danube tributaries (especially Inn, Mur, Enns and Lech) show little relief. Also the Durance in the Western Alps has little relief.

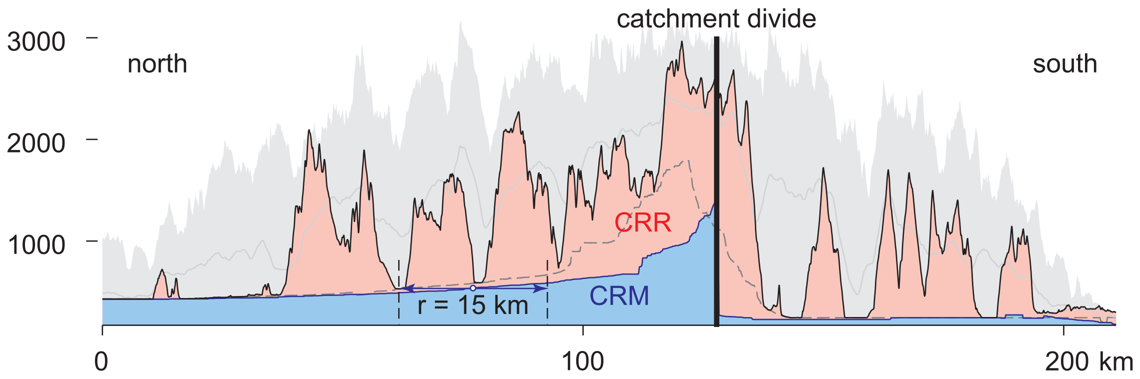

ESM 2 Fig 4: Profile of the Alps with CRR, CRM and swath
Location of the profile is shown in ESM 2 Fig. 3, it spans from Lindau to the Milan area. Topography is shown in black. Swath data is displayed in grey: Continous line is the average elevation, elevation range is shaded; dashed line is minimum elevation. The blue shading is the *Catchment-Restricted Minimum Elevation* (CRM) and the red above the CRM is the *Catchment-Restricted Relief* (CRR). Note that CRM is lower than the swath profile because it refers to a minimum in a circle and not only the line as in the swath. We display the 15 km search radius of CRM around a point of interest in profile direction to illustrate the difference. The catchment restriction is visible as a black separator.
