# Supplementary material for: Greater Alpine river network evolution, interpretations based on novel drainage analysis
Source: Swiss J Geosci. 2018 Nov 22;112(1):3–22. doi: 10.1007/s00015-018-0332-5 (PMC7081830; doi:10.1007/s00015-018-0332-5)

χ MAP OF THE ALPS

Sascha WINTERBERG

Sean WILLETT

Geological Institute, ETH Zürich

sascha.winterberg@erdw.ethz.ch

ETH zürich

Swiss Journal of Geosciences

Original map size is A1 or 84x60 cm.

Projected coordinate system (all maps)
WGS 1984 UTM Zone 32 N
Transverse Mercator

All coordinate units and elevations are given in meters.
Names are according to local language except for the overview map.

ALPINE χ MAP AND WINDGAPS

1:1'500'000

100 km

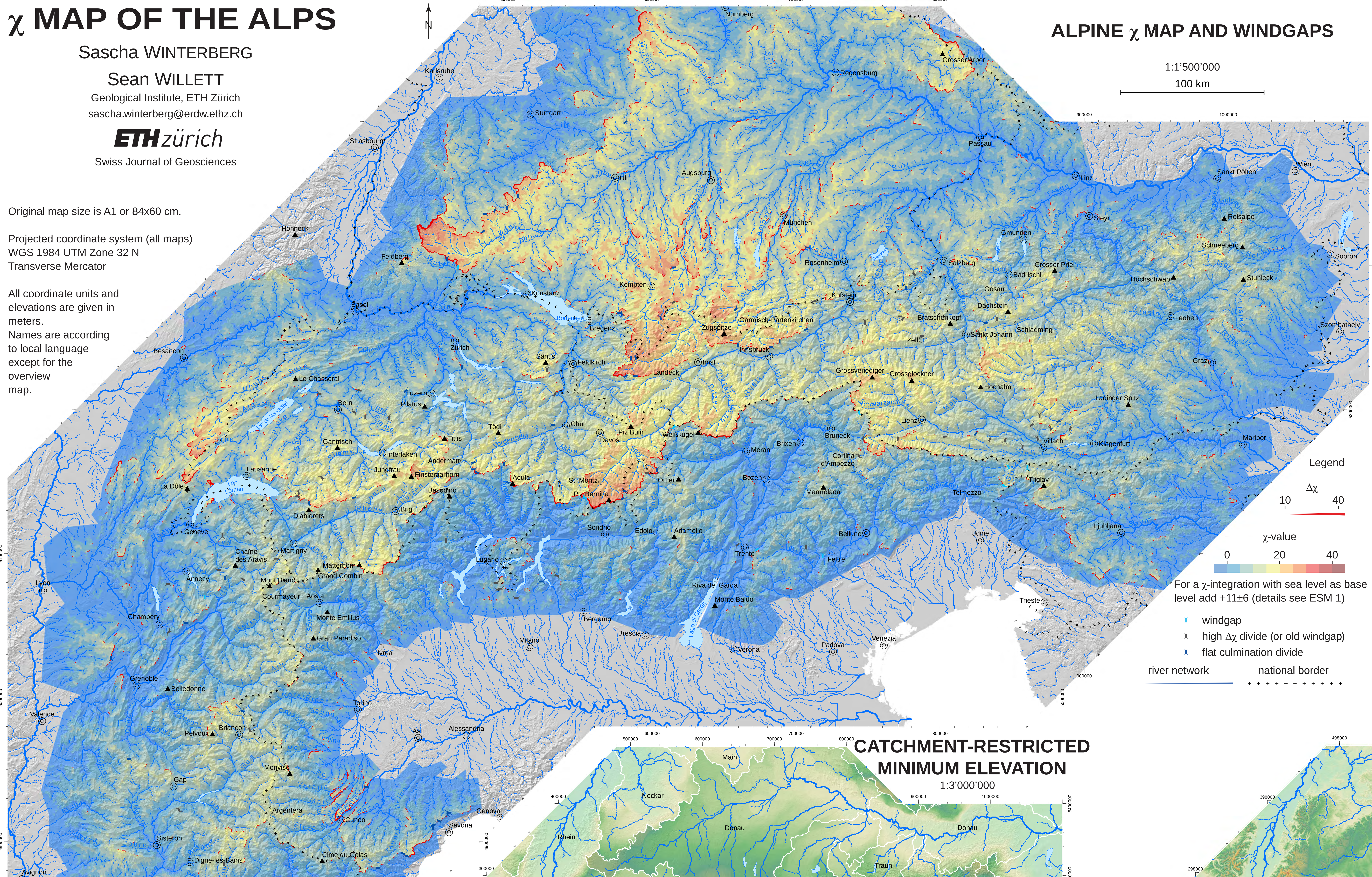

Overview

1:25'000'000

UTM 32 N

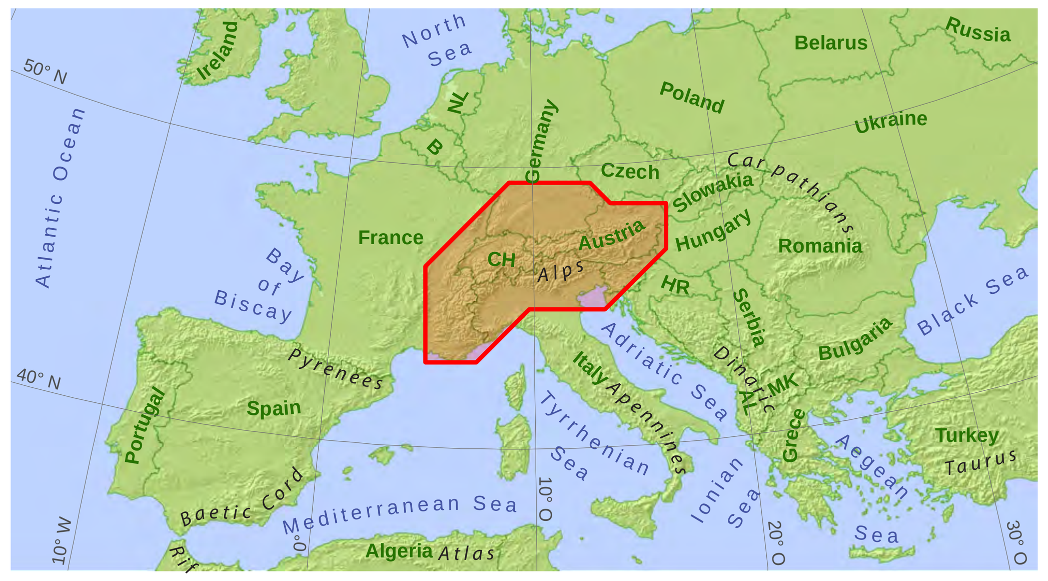

About the letter χ

χ refers to the 22nd letter of the Greek alphabet.
χ is written chi and pronounced ˈkaɪ or ˈki in English.

Interpretation of χ

Δχ values show the stability of a drainage divide. To restore steady state, river catchments with high χ-values have to loose catchment against rivers with low χ-values. This process happens either by continous migration of the divide or by river capture and subsequent instantaneous loss of catchment area.

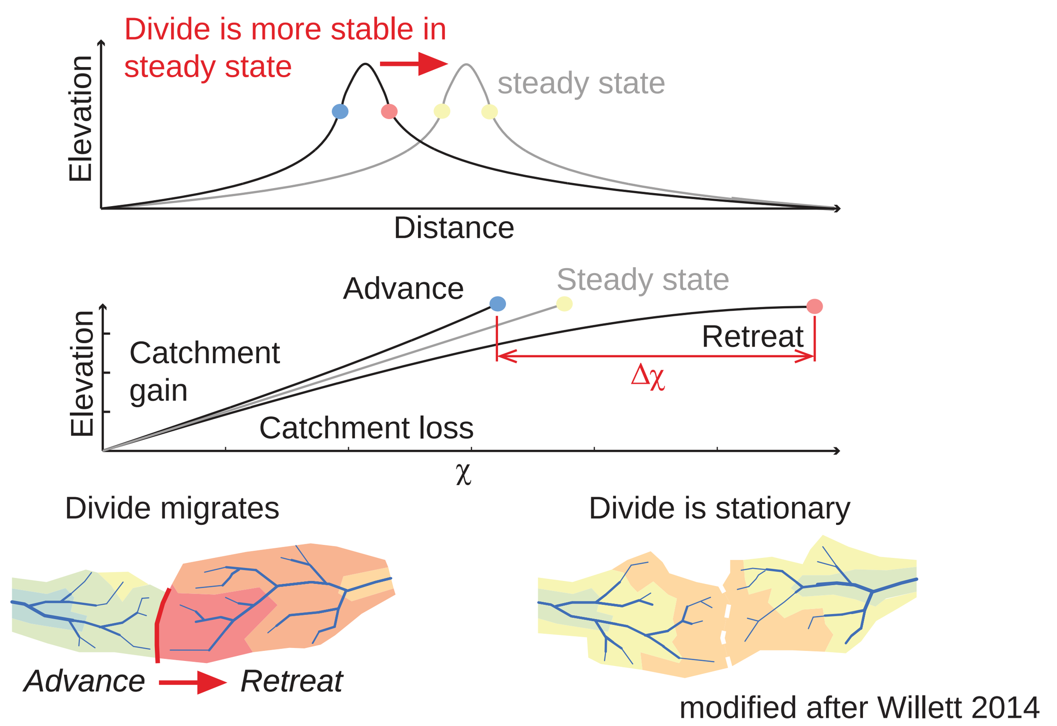

modified after Willett 2014

Calculation of χ

χ values are calculated as an integral of catchment area from a common base-level. Areas where hillslope processes dominate are excluded. The uphill area needed to form a stream is 10^6 m². Scaling area, A_0 is 1 m².

$$\chi = \int_{x_b}^{x} \frac{A_0}{Ax'}^{\frac{m}{n}} dx'$$

min. area streams $A_{min} = 10^6 \text{ m}^2$
distance from baselevel x ($z_b = 250$ m a.s.l.)
concavity $m/n = 0.45$

References

Fox, M., Herman, F., Willett, S.D. and Schmid, S.M.: The Exhumation history of the European Alps inferred from linear inversion of thermochronometric data, American Journal of Science

Willett, S. D., McCoy, S. W., Perron, J. T., Goren, L., and Chen, C.-Y., 2014, Dynamic reorganization of river basins: Science

Data sources maps

SRTM 3 arc second: USGS Earth Explorer (downloaded 2015)
National borders: Eurostat (CNTR 2014)
Ocean bathymetry: European Marine Observation and Data Network (EMOD)

Acknowledgements

Swiss NSF SINERGIA Swiss AlpArray (grant 154434)

CATCHMENT-RESTRICTED MINIMUM ELEVATION

1:3'000'000

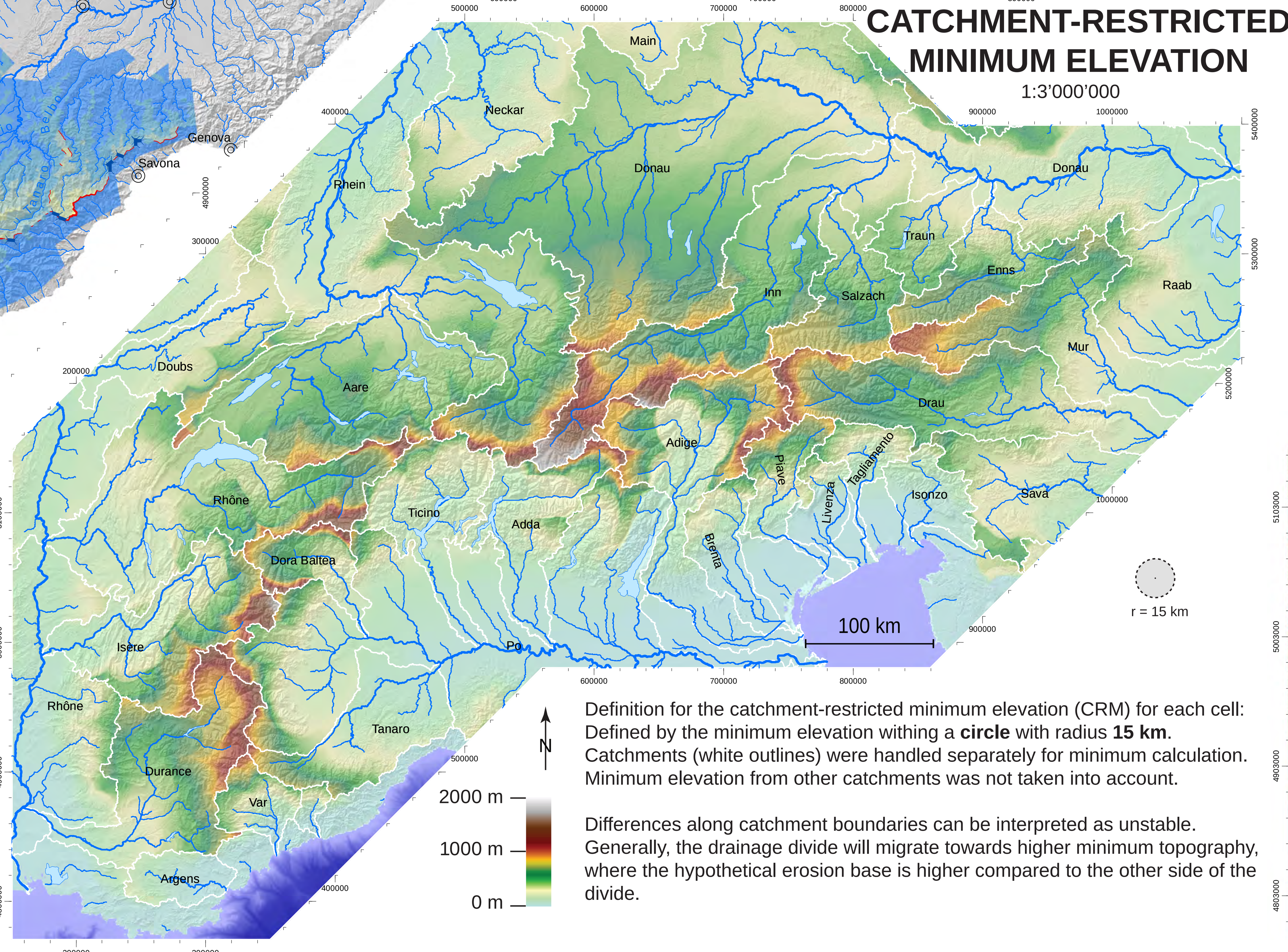

Definition for the catchment-restricted minimum elevation (CRM) for each cell: Defined by the minimum elevation withing a **circle** with radius **15 km**. Catchments (white outlines) were handled separately for minimum calculation. Minimum elevation from other catchments was not taken into account.

Differences along catchment boundaries can be interpreted as unstable. Generally, the drainage divide will migrate towards higher minimum topography, where the hypothetical erosion base is higher compared to the other side of the divide.

UPLIFT & EXHUMATION on topographic map

1:3'000'000

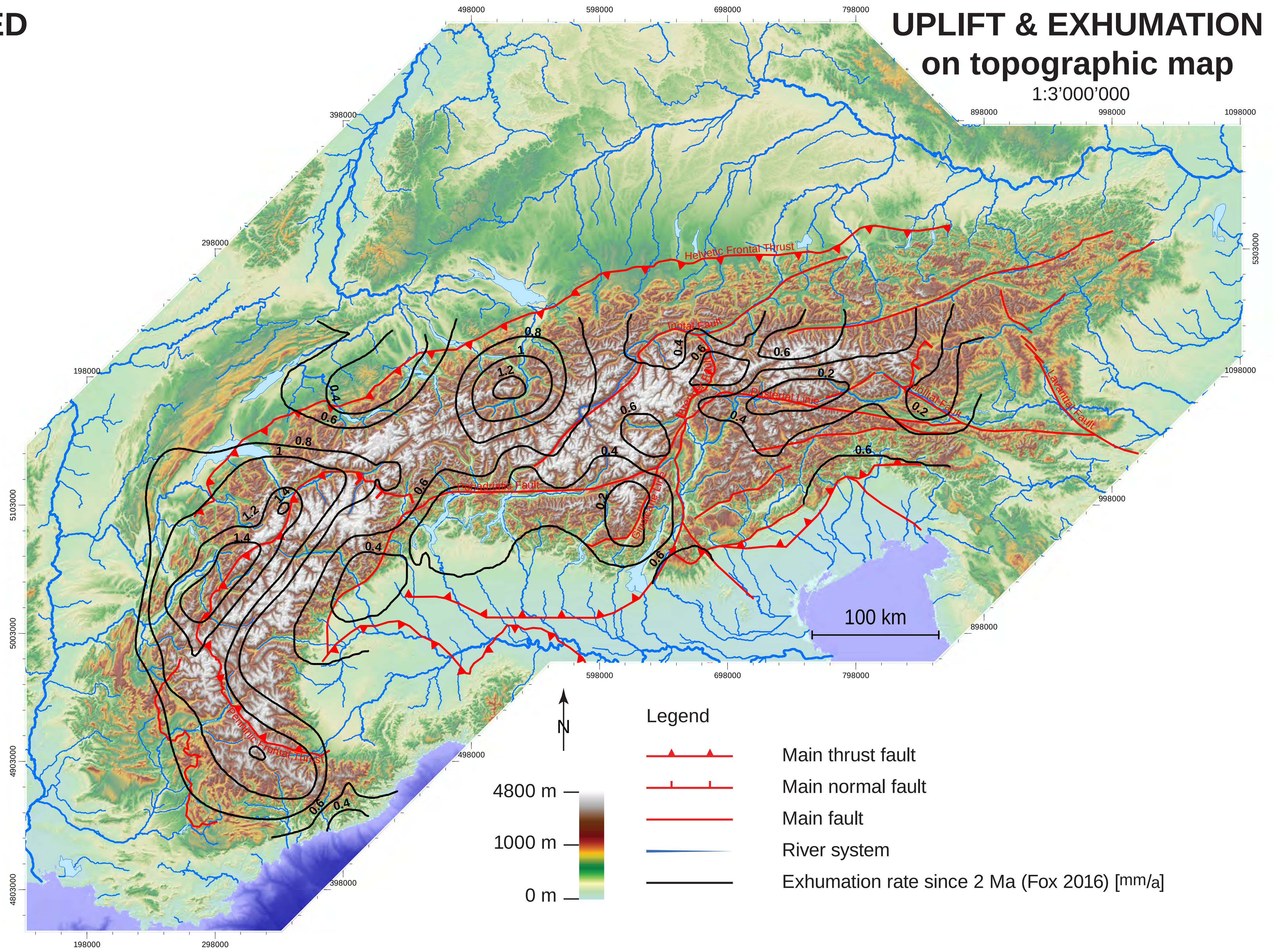

Supplement: Supplementary file 3 — Supplementary material 3 (PDF 9597 kb) [file 15_2018_332_MOESM3_ESM.pdf]
